# Using a comprehensive atlas and predictive models to reveal the complexity and evolution of brain-active regulatory elements

**DOI:** 10.1126/sciadv.adj4452

**Published:** 2024-05-23

**Authors:** Henry E. Pratt, Gregory Andrews, Nicole Shedd, Nishigandha Phalke, Tongxin Li, Anusri Pampari, Matthew Jensen, Cindy Wen, PsychENCODE Consortium, Michael J. Gandal, Daniel H. Geschwind, Mark Gerstein, Jill Moore, Anshul Kundaje, Andrés Colubri, Zhiping Weng

**Affiliations:** ^1^ Department of Genomics and Computational Biology, University of Massachusetts Chan Medical School, Worcester, MA 01605, USA.; ^2^ Khoury College of Computer Science, Northeastern University, Boston, MA 02115, USA.; ^3^ Department of Genetics, Stanford University School of Medicine, Stanford, CA 94305, USA.; ^4^ Department of Molecular Biophysics and Biochemistry, Yale University, New Haven, CT 06520, USA.; ^5^ Interdepartmental Program in Bioinformatics, University of California, Los Angeles, Los Angeles, CA 90095, USA.; ^6^ Department of Psychiatry, David Geffen School of Medicine, University of California, Los Angeles, Los Angeles, CA 90095, USA.; ^7^ Department of Human Genetics, David Geffen School of Medicine, University of California, Los Angeles, Los Angeles, CA 90095, USA.; ^8^ Department of Psychiatry, Perelman School of Medicine, University of Pennsylvania, Philadelphia, PA 19104, USA.; ^9^ Lifespan Brain Institute, The Children’s Hospital of Philadelphia, Philadelphia, PA 19104, USA.; ^10^ Program in Neurogenetics, Department of Neurology, David Geffen School of Medicine, University of California, Los Angeles, Los Angeles, CA 90095, USA.; ^11^ Institute of Precision Health, University of California, Los Angeles, Los Angeles, CA 90095, USA.; ^12^ Program in Computational Biology and Bioinformatics, Yale University, New Haven, CT 06520, USA.; ^13^ Department of Computer Science, Yale University, New Haven, CT 06520, USA.; ^14^ Department of Statistics and Data Science, Yale University, New Haven, CT 06520, USA.; ^15^ Department of Computer Science, Stanford University, Stanford, CA 94305, USA.

## Abstract

Most genetic variants associated with psychiatric disorders are located in noncoding regions of the genome. To investigate their functional implications, we integrate epigenetic data from the PsychENCODE Consortium and other published sources to construct a comprehensive atlas of candidate brain cis-regulatory elements. Using deep learning, we model these elements’ sequence syntax and predict how binding sites for lineage-specific transcription factors contribute to cell type–specific gene regulation in various types of glia and neurons. The elements’ evolutionary history suggests that new regulatory information in the brain emerges primarily via smaller sequence mutations within conserved mammalian elements rather than entirely new human- or primate-specific sequences. However, primate-specific candidate elements, particularly those active during fetal brain development and in excitatory neurons and astrocytes, are implicated in the heritability of brain-related human traits. Additionally, we introduce PsychSCREEN, a web-based platform offering interactive visualization of PsychENCODE-generated genetic and epigenetic data from diverse brain cell types in individuals with psychiatric disorders and healthy controls.

## Teaser

Integration of PsychENCODE data reveals brain regulatory syntax and mammalian evolution, accessible via the PsychSCREEN web tool.

## INTRODUCTION

The human brain, with its estimated 100 to 200 billion neurons and supporting glial cells (*1*), gives rise to the capacity for cognition, language, and abstract thought that makes our species unique. Yet, how these phenomena emerge from the brain and to what extent they truly are extraordinary in the animal kingdom remain topics of scientific investigation and debate (*2*). The brain’s anatomy and constituent cell types are encoded by the 3 billion base pairs (bp) of the human genome, more than 98% of which are noncoding. Brain-specific regulatory elements within this noncoding compartment, such as promoters, enhancers, repressors, and insulators, play key roles in the functions of neurons and glia by regulating neuron- and glia-specific transcriptional programs (*3*–*7*). A complete understanding of these regulatory elements is essential to understanding the brain’s physiology and its pathophysiology. Genome-wide association studies (GWAS) show that many brain-related traits, including intelligence, neuroticism, and insomnia, as well as various psychiatric disorders, including major depressive disorder, bipolar disorder, and alcohol use disorder, are at least partially heritable (*8*–*14*). Most genomic loci implicated by GWAS are noncoding, but they are enriched with putative noncoding regulatory elements (*15*, *16*), suggesting that these elements may play a key role in common psychiatric and neurologic traits. For some psychiatric traits, the implicated elements are known to be brain-specific: schizophrenia, for example, is highly heritable, with genetic predisposition accounting for an estimated 70 to 80% of trait variability, and schizophrenia-associated variants are strongly enriched within brain-specific enhancers (*17*, *18*).

Recognizing the importance of brain-active regulatory elements, multiple initiatives have been undertaken to build atlases of these elements (*3*, *19*–*25*). Widely used approaches for identifying regulatory elements include biochemical assays such as assay for transposase-accessible chromatin with sequencing (ATAC-seq) (*26*) and deoxyribonuclease sequencing (DNase-seq) (*27*), which profile chromatin accessibility, and chromatin immunoprecipitation followed by sequencing (ChIP-seq) (*28*–*30*), which maps histones bearing characteristic posttranslational modifications. Major efforts including the Roadmap Epigenomics Project and the ENCODE Project (*15*, *31*) have mapped hundreds of thousands of brain-active regulatory elements, predominantly in homogenized brain tissue from healthy individuals; however, resolving the cell type specificity of these elements and their dysregulation in psychiatric diseases remains challenging. The PsychENCODE Consortium has profiled the epigenetic and transcriptomic landscapes of samples from more than 2000 human brains, covering a variety of brain regions and pathophysiologic states, including autism spectrum disorder and schizophrenia, identifying tens of thousands of brain-active regulatory elements (*3*, *4*, *32*, *33*). PsychENCODE’s most recent phase (phase 2) offers an expanded analysis of the brain at the single-cell level and throughout fetal development. In addition, other studies have focused on mapping single-cell chromatin accessibility in the fetal and adult brain (*19*–*21*) and investigating how the regulatory landscape influences the transcriptome of developing human brains and differentiating neural progenitors (*22*–*24*).

Evolutionary conservation can be leveraged to identify regulatory sequences under selective pressure. Many loci, while strongly conserved across the mammalian lineage, have undergone bursts of changes since humans diverged from chimpanzees; these human-accelerated regions show neuron-specific enhancer activity and play roles in regulating neurodevelopmental gene expression (*34*–*36*). The Zoonomia Consortium recently sequenced genomes from 240 mammalian species, offering unprecedented resolution for studying mammalian evolution of functional elements. A neural network-based approach leveraged sequence conservation to predict chromatin accessibility across species; this approach linked tens of thousands of cortical regulatory elements with the variation in mammalian brain size (*37*). In agreement with the aforementioned findings (*34*–*36*), regulatory elements near brain-relevant genes were found to have originated early in mammalian evolution and have subsequently experienced substantial sequence turnover (*38*). Last, evolutionary conservation and machine learning approaches can link brain trait heritability to both regulatory effects and candidate molecular effects, such as transcription factor (TF) binding (*19*, *38*).

Here, we integrate results from the first and second phases of the PsychENCODE Project and the ENCODE project to assemble a comprehensive atlas of candidate brain-active cis-regulatory elements, which we call b-cCREs. Leveraging chromatin accessibility data from adult and fetal brains, fluorescence-activated nucleus (FAN)–sorted neurons and glia, and single-cell ATAC-seq (scATAC-seq) datasets, we annotate subsets of b-cCREs according to the developmental time points at which they are active and the cell types in which they most likely function. We then train several computational models to understand differences in sequence syntax driving b-cCRE activity in different brain cell types and developmental time periods and integrate these with single-cell RNA-seq (scRNA-seq) data from PsychENCODE to predict which TFs might drive these differences. Last, we analyze 87 brain-related GWAS to understand how trait heritability is related to the regulatory function of b-cCREs. We make these analyses, as well as the PsychENCODE data from which they derive, available through a web-based visualization platform, PsychSCREEN.

## RESULTS

### Chromatin accessibility identifies candidate fetal and adult brain-active regulatory elements

Our primary goal is to provide a comprehensive resource for understanding gene regulation in the human brain, both during development and in adulthood. To this end, we constructed a comprehensive atlas of b-cCREs, which we call b-cCREs because they represent a subset of the latest version of roughly 2.35 million nucleosome-resolution candidate cis-regulatory elements (cCREs), which our group built for the ENCODE Project (*15*). We estimate that the cCREs, which integrate results from more than 1300 DNase-seq experiments and more than 1500 ChIP-seq experiments, account for the vast majority of cis-regulatory information in the human genome (*15*).

**Fig. 1.**
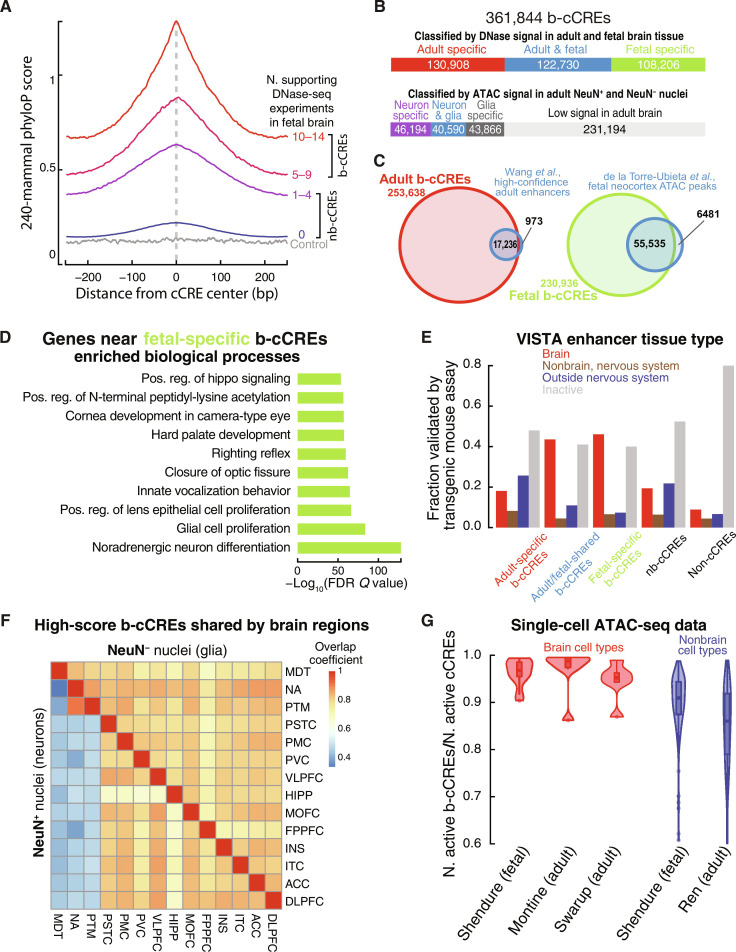
Construction and characteristics of our PsychENCODE atlas of b-cCREs. (**A**) 240-mammal phyloP scores of ENCODE cCREs, binned by the number of fetal brain DNase-seq experiments that support the cCRE’s activity. cCREs active in five or more DNase experiments (pink and red) are defined as fetal b-cCREs, while cCREs active in one to four biosamples (purple) are not unless they have DNase signal in the 99th percentile. These contrast cCREs that are not brain active (blue), and 10,000 dinucleotide matched random genomic regions (gray). (**B**) Stacked bar charts representing the classification of b-cCREs based on identification in fetal-specific (green), adult-specific (red), or both fetal and adult biosamples (blue) (top) and the classification of b-cCREs as neuron-specific (purple), glia-specific (gray), neuron and glia (blue), or low signal (light gray) based on adult NeuN FAN-sorted ATAC data (bottom). (**C**) Venn diagrams representing the overlap between adult b-cCREs and a published high-confidence enhancer set in adult brain (left) and between fetal b-cCREs and a published set of ATAC-seq peaks from the developing human cerebral cortex (right). (**D**) Ten most significantly enriched biological processes from GREAT analysis of fetal-specific b-cCREs. FDR, false discovery rate. (**E**) The validation rate of b-cCREs by transgenic mouse assays in the VISTA database. Fractions of b-cCREs, nb-cCREs, and non-cCREs overlapping VISTA enhancers that are active in various tissue types are grouped by color. (**F**) Overlap of active b-cCREs in 14 different brain regions (table S1D). The top-right and bottom-left triangles show pairwise overlap coefficients between different brain regions for NeuN^−^ and NeuN^+^ nuclei, respectively. (**G**) b-cCRE activity, represented by the proportion of active b-cCREs over active cCREs, in brain (red) and nonbrain (blue) scATAC-seq experiments. Each violin plot represents a different single-cell study, with each point being a cell type from pseudo-bulk scATAC-seq data.

We used a consensus approach of defining b-cCREs as cCREs that are chromatin-accessible in a minimum number of adult and fetal brain DNase-seq experiments (see Materials and Methods). We considered 96 ENCODE DNase-seq experiments from healthy adult brain tissue and 14 from fetal brain tissue in this analysis (table S1, A and B). These biosamples were taken from a variety of different brain regions, both cortical and subcortical. We evaluated sequence conservation of the cCREs supported by varying numbers of DNase-seq experiments at two evolutionary distances—the phyloP scores across 240 mammalian species (240-mammal phyloP) and the phastCons scores across the subset of 43 primate species (43-primate phastCons) surveyed by the Zoonomia consortium (*37*, *39*). Overall, cCREs supported by any number of brain DNase-seq experiments (with DNase signal above the 95th percentile in an experiment, see Materials and Methods) are more conserved in mammals and primates than those not (Fig. 1A and fig. S1, A to D). We defined adult b-cCREs as the subset of cCREs with support from at least five adult brain DNase-seq experiments because they have higher conservation than the cCREs with support from fewer experiments, with subsequent diminishing returns for additional supporting experiments (fig. S1, A and B). We applied the same cutoff of 5 for fetal brain DNase experiments to define fetal b-cCREs because they show the same trend of evolutionary conservation (Fig. 1A and fig. S1, C and D). Because there are fewer fetal DNase-seq experiments available, we further expanded the fetal set of b-cCREs to include all cCREs with a DNase signal above the 99th percentile in at least one fetal dataset, using a stricter percentile to avoid false negatives. We ultimately arrived at a combined set of 361,844 b-cCREs in adult and fetal brains (Fig. 1B, top, and table S2). For brevity, the cCREs that are not b-cCREs are called nb-cCREs (*N* = 1,987,010).

There are substantial differences between the regulatory landscapes of the adult and fetal brains: 130,908 b-cCREs are likely specifically active in the adult brain, 108,206 specifically in the fetal brain, and 122,730 in both adult and fetal brains (Fig. 1B, top). We performed gene ontology (GO) analysis on these subsets using the Genomic Regions Enrichment of Annotations Tool (GREAT) (*40*). Adult b-cCREs are near genes such as *SCYL1* (SCY1-like pseudokinase 1) and *BAIAP2* (BAR/IMD domain containing adaptor protein 2), which are involved in maintaining mature neuronal function (*41*, *42*). However, many immune genes and GO terms are highly enriched in adult b-cCREs (fig. S2A and table S3A). Several genes involved in neurodevelopment are near fetal-specific b-cCREs, such as SRY-box TFs 4 and 11—*SOX4* and *SOX11*—and activator of transcription and developmental regulator *AUTS2* (*43*–*45*). In addition, the most significantly enriched GO biological processes for fetal b-cCREs are related to the development and differentiation of neurons and glia, as well as the development of the eyes and mouth (fig. S2B and table S3B).

We next compared our fetal and adult sets of b-cCREs with existing brain-active regulatory element collections. Our adult-specific set nearly perfectly recapitulates a set of approximately 18,000 high-confidence adult brain enhancers published by Gerstein and colleagues (*3*) during the first phase of PsychENCODE but substantially expands its comprehensiveness (Fig. 1C, left). Likewise, our fetal-specific set recapitulates and expands upon a set of 61,000 fetal neocortex ATAC-seq peaks (Fig. 1C, right) (*21*). We also examined the evolutionary conservation of fetal- and adult-specific b-cCREs, revealing that fetal-specific b-cCREs are more conserved than adult-specific b-cCREs across the 240 mammals and the subset of 43 primates, while adult/fetal-shared b-cCREs are as conserved as fetal-specific b-cCREs (fig. S1, E and F). We then compared b-cCREs against an atlas of putative enhancers active during fetal brain development obtained by meta-analysis of epigenetic data at five distinct developmental time points (*25*). That catalog included 202,124 elements, of which 39,705 exhibited differential epigenetic signals across development—the authors termed the subset “differentially active elements” (DAEs), and their analysis indicated that DAEs were more likely to be functional in the developing brain than the remaining elements (nDAEs). Our b-cCREs recapitulate a majority of the DAEs (22,524; 56.7%) but a lower percentage (18.3%; *N* = 29,736) of the nDAEs. Furthermore, as would be expected given their definition, our fetal-specific and adult/fetal-shared b-cCREs are far more likely than adult-specific b-cCREs to intersect DAEs (fig. S3A). Notably, most of the DAEs not captured by our b-cCRE catalog (83.4%; *N* = 14,339) are nb-cCREs. On average, DAEs that intersect b-cCREs exhibit higher average chromatin accessibility and surrounding levels of H3K27ac and H3K4me1 (histone modifications indicative of enhancers) in fetal brain samples (fig. S3, B to D, and table S1C). Furthermore, the DAEs and nDAEs that intersect b-cCREs are more evolutionarily conserved than those that do not (fig. S3, E and G), with the portions that overlap b-cCREs being even more conserved than the non-overlapping regions (fig. S3, F and H). In other words, DAEs that are also b-cCREs contain a particularly high-confidence subset of fetal brain enhancers with additional lines of evolutionary and epigenetic support.

**Fig. 2.**
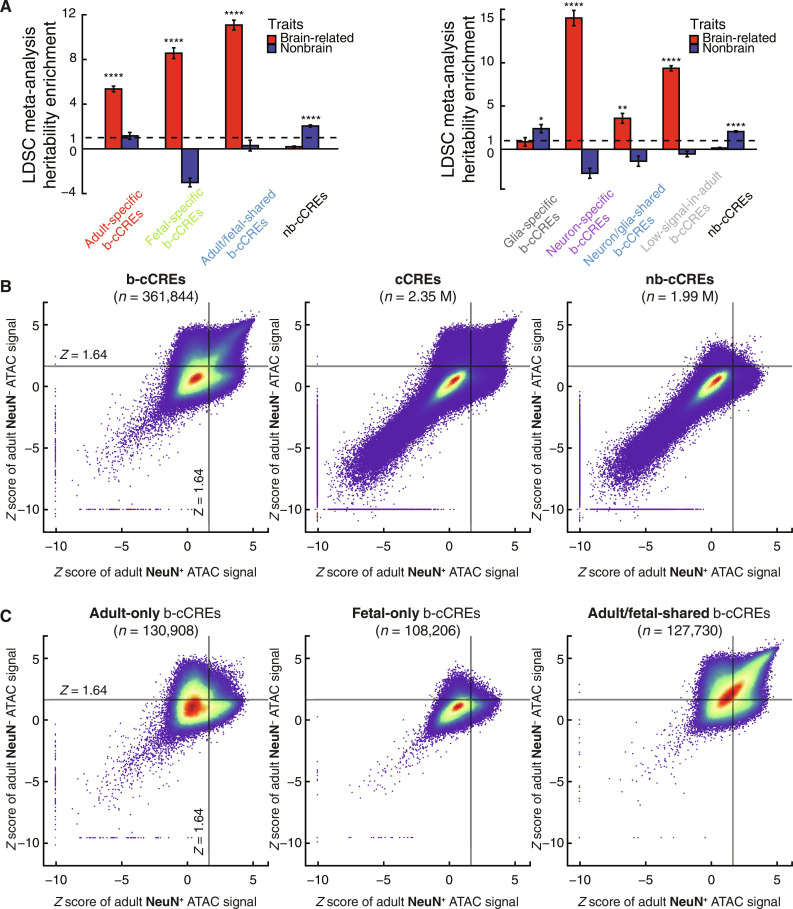
Identifying adult-specific, fetal-specific, neuron-specific, and glia-specific b-cCREs and characterizing their role in complex traits. (**A**) Heritability enrichment meta-analysis of adult-specific, fetal-specific, shared b-cCREs, and nb-cCREs (left) and neuron-specific, glia-specific, neuron/glia-shared b-cCREs, b-cCREs with low signal in both NeuN^+^ and NeuN^−^ adult samples, and nb-cCREs (right) in brain-related traits (red) and nonbrain-related traits (blue). LDSC meta-analysis *P* value for enrichment in heritability of genetic variants residing in subsets of b-cCREs: *P < 0.05, **P < 0.01, and ****P < 0.0001. (**B**) Heatscatter comparing *z* scores of b-cCREs (left-most plot) from NeuN^+^ versus NeuN^−^ ATAC-seq data aggregated across multiple donors. Black lines intersecting the plot represent a *z* score of 1.64, our threshold of defining an active cCRE. Middle and right-most plots are the same but with all cCREs (middle) and nb-cCREs (right). (**C**) Same as (B) but plotting adult-specific b-cCREs (left), fetal-specific b-cCREs (middle), and b-cCREs active in both adult and fetal biosamples (right).

To further validate our consensus approach of defining b-cCREs, we assessed the heritability (h^2^) enrichment of genetic variants within b-cCREs for complex traits with partitioned linkage disequilibrium (LD) score regression (LDSC) (*46*, *47*) . We assembled a collection of 204 GWAS summary statistic sets from the LDSC authors and the Psychiatric Genomics Consortium, of which 87 are from brain-related traits and 117 nonbrain-related traits (table S4A). We compared the heritability enrichment of b-cCREs against the cCREs predicted to be active using individual ENCODE DNase-seq experiments (the aforementioned 96 experiments on adult brain tissues and 14 on fetal brain tissues; table S1, A and B). We defined one set of active cCREs per experiment (in total 96 and 14 individual experiment–active cCRE sets for adult and fetal brains, respectively), which were used to define b-cCREs in our consensus approach, as described above (see Materials and Methods for more details). The b-cCRE set displays a greater enrichment than the individual experiment–active cCRE sets for the vast majority of brain-related traits. In particular, the b-cCRE set shows a greater enrichment than the median enrichment of the individual experiment–active cCRE sets for all brain-related traits (fig. S4, A and B, and table S4, C and D). This suggests that our consensus methodology identifies cis-regulatory elements likely to affect the heritability of psychiatric diseases and other brain-related traits. We also performed LDSC meta-analyses (*46*) on the heritability enrichment of b-cCRE subsets across the 87 brain traits and, separately, across the 117 nonbrain traits. Overall, the brain-related traits display significantly higher heritability enrichment within b-cCREs than the nonbrain-related traits; adult/fetal-shared b-cCREs display the strongest enrichment across the brain-related traits, followed by fetal-specific b-cCREs and then adult-specific b-cCREs (Fig. 2A, left, and table S4E; 11.08-fold; enrichment *P* = 9.97 × 10^−34^, 8.56-fold; *P* = 6.51 × 10^−26^, and 5.36-fold; *P* = 8.33 × 10^−17^, respectively). Notably, b-cCREs do not show significant enrichment for nonbrain-related traits, while nb-cCREs show significant enrichment only for nonbrain-related traits (2.05-fold; *P* = 4.49 × 10^−23^, Fig. 2A, left).

We note that the psychiatric traits in this panel display higher genetic correlation with one another than the nonpsychiatric traits. To ensure that our results were not biased by genetic correlation, we repeated the analysis on a subset of 81 traits from this panel (34 brain-related and 47 nonbrain-related), all of which have squared genetic correlation less than 0.1 with each other (table S4, A and B), as previously described (*48*). The overall findings are consistent (fig. S4C and table S4F).

### Subsets of b-cCREs exhibit cell type– and brain region–specific chromatin accessibility and enhancer activity in transgenic mouse assays

To determine whether our annotations of b-cCREs based on their tissue-specific chromatin accessibility were predictive of brain-specific regulatory activity, we compared our b-cCREs with the VISTA collection of in vivo functional testing data (*49*–*51*). The latest release of the VISTA resource comprised 2002 distinct human genomic regions tested for enhancer activity in transgenic mouse embryos. Overall, 2926 b-cCREs (1398 fetal-specific, 508 adult-specific, and 1020 fetal/adult-shared) and 4757 nb-cCREs overlapped VISTA regions, which tend to be larger than cCREs. We found that 46.1% of tested fetal-specific b-cCREs demonstrated enhancer activity specifically in embryonic brain according to transgenic mouse experiments, with a further 6.6% validating in nonbrain embryonic tissues in the nervous system (e.g., cranial nerve) and 7.4% validating in other non-nervous system tissues; 40.0% tested negative (Fig. 1E and table S1G). The validation rate was slightly lower for adult/fetal-shared b-cCREs (43.5%). In contrast, adult-specific b-cCREs validated in brain at a significantly lower rate (18.1%, Χ^2^ test *P* = 2.4 × 10^−4^ versus fetal-specific b-cCREs), consistent with developmental stage of the transgenic mice. We also observed a significant lower validation rate for nb-cCREs than fetal-specific b-cCREs (19.4%; *P* = 1.5 × 10^−19^). Few tested VISTA regions outside cCREs (non-cCREs) demonstrated enhancer activity in brain tissues (8.9%; Χ^2^ test *P* = 3.7 × 10^−7^ versus fetal-specific b-cCREs).

PsychENCODE has produced ATAC-seq data in FAN-sorted neurons [positive for the neuronal marker NeuN (NeuN^+^)] and glia (NeuN^−^) from a variety of adult postmortem brain regions, both cortical and in the basal ganglia, enabling us to classify b-cCREs according to their cell type and brain region specificity (*6*, *52*). In addition, we leveraged publicly available scATAC-seq datasets to understand the activity profile of b-cCREs in various glial and neuronal subtypes (*19*, *20*, *53*, *54*).

We analyzed the similarity of b-cCRE landscapes in FAN-sorted NeuN^+^ nuclei and FAN-sorted NeuN^−^ nuclei across 62 specimens—51 from cortical brain regions and 11 from subcortical brain regions, including 5 from the basal ganglia (table S1D). Active b-cCREs were identified for each ATAC-seq experiment (see Materials and Methods). Then, we computed the pairwise overlap coefficients of active b-cCREs for all glial experiment pairs and all neuronal experiment pairs (fig. S5). To simplify the comparison between different brain regions, we combined the NeuN^+^ experiments in the same brain region to call a set of active b-cCREs (see Materials and Methods), applying the same approach to NeuN^−^ experiments. Subsequently, we computed overlap coefficients between brain regions (Fig. 1F). Our results indicated high overlaps among all glial experiment pairs, regardless of the brain region (Fig. 1F and fig. S5, top-right triangle). In contrast, the neuronal experiments revealed two distinct clusters of b-cCRE similarity: Neurons from various cortical regions share high overlap in b-cCRE usage with one another, while neurons in subcortical regions, specifically those in the basal ganglia such as the putamen and nucleus accumbens, also showed high overlap. However, there was lower similarity when comparing the two clusters (Fig. 1F and fig. S5, bottom-left triangle). This pattern likely reflects differences in the regulatory landscape of the inhibitory medium spiny neurons in the basal ganglia compared with the landscape of excitatory pyramidal neurons in the cerebral cortex.

We then analyzed the b-cCRE landscapes of cell types identified by four scATAC-seq studies (*19*, *20*, *53*, *54*). For each cell type, we compared the active b-cCREs and active cCREs, defined using the pseudo-bulk ATAC-seq signal of the respective cell type (see Materials and Methods). In total, we examined 25 cell types in the brain and 174 cell types in other tissues across these studies, maintaining a distinction between identical cell types defined in multiple studies. Active b-cCREs constituted more than 90% of active cCREs in all but two of the brain cell types. Notably, these exceptions were both microglia, the resident immune cells of the brain, as defined by two separate studies. The median percentage of active cCREs being b-cCREs exceeded 95% for brain cell types in all studies. This extent of overlap was significantly greater than that observed in nonbrain cell types from the same studies (Wilcoxon rank sum test *P* = 1.17 × 10^−9^; Fig. 1G), suggesting that our b-cCRE catalog comprehensively captures regulatory elements that function in the brain.

### Neuron-specific and glia-specific b-cCREs display differences in evolutionary conservation and contribution to complex human traits

Motivated by our above analysis of cell type specificity, we classified b-cCREs into three groups: neuron-specific, glial-specific, and neuron/glial-shared. This classification was based on the ATAC-seq signal (normalized and expressed in *z* score) across all adult FAN-sorted NeuN^+^ nuclei and NeuN^−^ nuclei from PsychENCODE (see Materials and Methods) (table S2A). When b-cCREs are plotted on the *x*-*y* plane according to these *z* scores, they exhibit three distinct clusters of enrichment: one showing high *z* scores in both neurons and glia, one with a high *z* score only in neurons, and one with a high *z* score only in glia (Fig. 2B, compare the left with the middle). In contrast, nb-cCREs appear relatively depleted in all three clusters (Fig. 2B, right). On this basis, we defined neuron-active b-cCREs as having high NeuN^+^ *z* scores and glial-active b-cCREs as having high NeuN^−^ *z* scores (see Materials and Methods). In total, we identified 46,194 neuron-specific b-cCREs, 43,866 glia-specific b-cCREs, and 40,590 neuron/glia-shared b-cCREs; the remaining 231,194 b-cCREs, which include 94,753 of the 108,206 fetal-specific b-cCREs, are classified as low-signal according to the current PsychENCODE ATAC-seq data on FAN-sorted cells from adult brains (Fig. 1B, bottom, and table S2B).

To further assess their cell type specificity, we compared these subsets of b-cCREs with two complementary regulatory annotations distinct from chromatin accessibility. We first intersected the b-cCREs with gene enhancer loops and frequently interacting regions (FIREs) derived from Hi-C data in FAN-sorted neurons and glia from adult brain (*55*). As compared with permuted sets of control cCREs and randomly selected genomic regions (see Materials and Methods), our neuron-specific b-cCREs are strongly enriched within neuron-specific FIREs and enhancer-promoter loops but not glia-specific FIREs or enhancer-promoter loops (fig. S6, A and B). Likewise, our glia-specific b-cCREs are strongly enriched within glia-specific FIREs and enhancer-promoter loops but not neuron-specific FIREs or enhancer-promoter loops (fig. S6, A and B). Second, we compiled a list of cell type–specific marker genes from the literature based on brain scRNA-seq data (table S1E) and assessed the enrichment of b-cCRE subsets in proximity (within 100 kb; see Materials and Methods) to these genes. Neuron-specific b-cCREs exhibit the strongest enrichment proximal to excitatory neuron marker genes, while glia-specific b-cCREs exhibit enrichment proximal to astrocyte, oligodendrocyte, oligodendrocyte precursor cell (OPC), and microglia marker genes (fig. S6C). These patterns align with brain development stages, where neurogenesis precedes gliogenesis, and myelination extends into at least the third decade of human life (*56*).

We also examined the evolutionary conservation of neuron- and glia-specific b-cCRE subsets. Neuron/glia-shared b-cCREs are the most conserved among mammals, evidenced by their, on average, higher phyloP scores across 240 mammals when compared with neuron-specific and glia-specific b-cCREs (fig. S1G). However, within the primate lineage, neuron-specific b-cCREs demonstrate the greatest conservation. They show, on average, higher phastCons scores across 43 primates than both glia-specific and shared b-cCREs (fig. S1H).

Expectedly, our collection of fetal-specific b-cCREs has relatively low ATAC-seq signals in both adult neurons and adult glia (Fig. 2C, middle, and table S2). Many adult-specific b-cCREs tend to be neuron-specific or glia-specific, while adult/fetal-shared b-cCREs tend to be neuron/glia-shared (Fig. 2C, left and right, and table S2). We performed GREAT analysis on the neuron-specific and glia-specific subsets, revealing distinct ontologies associated with each subset. Genes such as gamma-aminobutyric acid type B receptor subunit 2 (*GABBR2*), glutamate ionotropic receptor NMDA type subunit 2B (*GRIN2B*), and potassium calcium-activated channel subfamily M regulatory beta subunit 2 (*KCNMB2*), which are associated with neuronal development and function, are near neuron-specific b-cCREs (fig. S2C and table S3C) (*57*–*59*). Glia-specific b-cCREs are near genes such as RE1 silencing TF (*REST*) and interferon regulatory factor 2 (*IRF2*), which are often related to nonneuronal or immune function in the brain (fig. S2D and table S3C) (*60*, *61*). These nearby genes also enrich for relevant biological processes, such as axon generation or neurotransmitter signaling for neuron-specific b-cCREs and granule cell differentiation in glia-specific b-cCREs (fig. S2, C and D, and table S3D).

We next performed LDSC meta-analysis (*46*) to determine the relative contribution of neuron-specific, glia-specific, and neuron/glia-shared b-cCREs to complex traits using the same panel of GWAS described above (Fig. 2A, right). We observe significant enrichment for all three categories, but, notably, we find that the neuron-specific b-cCREs exhibit by far the highest enrichment for brain-related traits (15.17-fold; enrichment *P* = 2.85 × 10^−27^). The heritability enrichment for brain-related traits observed for neuron/glia-shared b-cCREs is less significant (3.58-fold; enrichment *P* = 7.52 × 10^−3^), and no enrichment was observed for glia-specific b-cCREs. In contrast, the glia-specific subset exhibited only modest enrichment for nonbrain traits (2.38-fold; enrichment *P* = 4.25 × 10^−2^), while the neuron-specific and neuron/glia-shared b-cCREs showed depletion for nonbrain traits. Meanwhile, the b-cCREs with low signals in PsychENCODE ATAC-seq data on adult brain samples (low-signal b-cCREs), which include most fetal-specific b-cCREs, also show significant enrichment for brain-related traits (Fig. 2A, right, and table S4G).

**Fig. 3.**
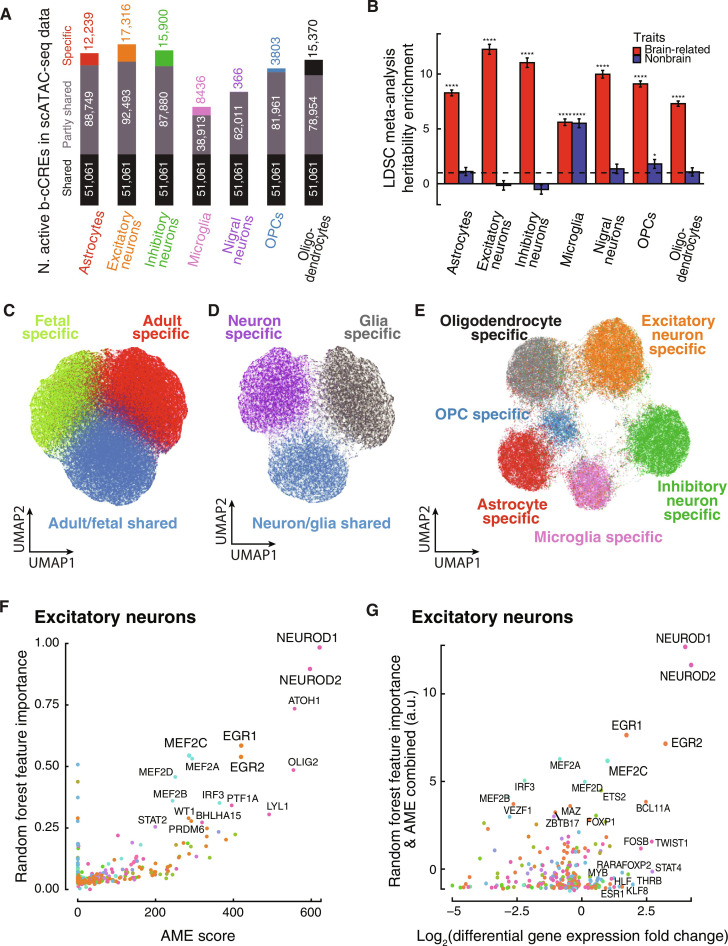
Machine learning of sequence features distinguishing b-cCREs active at different developmental time points and in different brain cell types. (**A**) Stacked bar charts representing the classification of b-cCREs based on identification in Corces *et al.* (*19*) single-cell ATAC cell types. Each bar represents the number of active b-cCREs within a particular cell type, split by activity in all seven cell types (black), activity shared among two to six cell types (gray), and activity specific to a particular cell type (colors vary by cell type). (**B**) Meta-analysis on the heritability enrichment in brain-related traits (red) and nonbrain-related traits (blue) for b-cCREs predicted to be active in each cell type using the Corces *et al.* (*19*) scATAC-seq data. LDSC meta-analysis *P* value for enrichment in heritability of genetic variants residing in subsets of b-cCREs: *P < 0.05 and ****P < 0.0001. (**C**) UMAP plot of latent sequence features learned by a variational autoencoder (VAE) within adult-specific (red), fetal-specific (green), and adult/fetal shared (blue) b-cCRE subsets. (**D**) UMAP plot of latent sequence features learned by a VAE within neuron-specific (purple), glia-specific (gray), and neuron/glia-shared (blue) b-cCRE subsets. (**E**) UMAP plot of latent sequence features learned by a VAE within cell type–specific b-cCRE subsets: Excitatory neurons (orange), inhibitory neurons (green), microglia (pink), astrocytes (red), oligodendrocytes (gray), and OPCs (blue). (**F**) Comparison of Analysis of Motif Enrichment (AME) scores (*x* axis) and random forest feature importance (*y* axis), to identify TF motifs that are found within excitatory neuron-specific b-cCREs, colored by TF family. (**G**) Differential gene expression of excitatory neurons versus all other cell types (*x* axis) is plotted against sum of scaled random forest feature importance and AME scores within excitatory neuron-specific b-cCREs using arbitrary units (a.u.) (*y* axis), colored by TF family.

To further explore the above results, we identified b-cCREs specifically active in several glial and neuronal cell types identified from the aforementioned scATAC-seq studies with brain cell types (table S1F) (*19*, *20*, *54*). We primarily used the data by Corces *et al.* (*19*), which identified seven cell types—astrocytes, excitatory neurons, inhibitory neurons, microglia, nigral neurons, oligodendrocytes, and OPCs. We identified the b-cCREs likely to be active in each of these cell types based on pseudo-bulk ATAC-seq signals (see Materials and Methods) (Fig. 3A and table S2, A and B). Related single-cell types share active b-cCREs to larger extents than do less related cell types (fig. S7A). We then performed LDSC meta-analysis and obtained highly consistent results across all brain scATAC-seq datasets, with significant heritability enrichment for brain-related traits observed for all the glia and neurons (Fig. 3B, fig. S7B, and table S4, H to J). In all cases, we note that enrichment for the neuronal cell types is significantly greater than that for the glial cell types, consistent with our above results in bulk neuron (NeuN^+^) and glial (NeuN^−^) cells. Notably, none of the single-cell types except for microglia and vascular endothelial cells exhibited high enrichment for nonbrain traits. Microglia share regulatory elements with other immune cell types; accordingly, the microglial b-cCRE heritability enrichment for nonbrain traits was derived predominantly from immune-mediated traits.

Similarly to NeuN^+^ and NeuN^−^ specific b-cCREs, cell type–specific b-cCREs are frequently found in proximity to cell type–specific marker genes and TFs (fig. S6D). Across the single-cell datasets, we consistently observed enrichment of excitatory neuron-, inhibitory neuron-, astrocyte-, oligodendrocyte-, OPC-, and microglia-specific b-cCREs within 100 kb of the corresponding cell type’s marker genes as defined based on literature annotations of scRNA-seq data (table S1E). Corces *et al.* (*19*) used chromatin accessibility near a limited number of marker genes to define scATAC-seq cell types, and removing these genes from the analysis still yielded consistently high enrichment in the corresponding cell types. Cell type–specific b-cCREs also display significant enrichment for biological processes relevant to their cell type, based on GREAT analysis (fig. S2, E to J, and table S3, E to J).

### Differences in sequence syntax define neuronal versus glial b-cCREs

The activities of cis-regulatory elements are mediated by TFs, proteins that recognize short characteristic motifs within functional DNA sequences. We aimed to understand how neuron-specific and glia-specific b-cCREs differ in the sequence syntax they use; this in turn sheds light on differences in TF usage between the cell types. To visualize this, we trained a variational autoencoder (VAE) on distinct classifications of b-cCREs. The encoder of the VAE transforms the b-cCREs into a lower dimension latent space, while the decoder reconstructs the original input from this minimal representation. A feed-forward neural network simultaneously uses the latent space to classify b-cCREs based on provided categories, i.e., neuron-specific, glia-specific, or neuron/glia-shared and fetal-specific, adult-specific, or adult/fetal-shared (see Materials and Methods).

To visualize the latent space of each model, we performed Uniform Manifold Approximation and Projection (UMAP) analysis (*62*, *63*) on the latent features. In theory, the two UMAP dimensions represent combinations of latent sequence features which are most effective at distinguishing different categories of b-cCREs. We find that adult-specific, fetal-specific, and adult/fetal-shared b-cCREs occupy distinct clusters when they are projected into UMAP space using the VAE’s latent encoding (Fig. 3C), suggesting that the model has learned sequence features to distinguish them. We were also able to train a separate model on the neuron-glia axis of b-cCREs, which exhibited similar class separation in the UMAP space (Fig. 3D), and another model on the six cell types identified by the Corces *et al.* (*19*) scATAC-seq data (Fig. 3E). We excluded nigral neurons from these and subsequent sequence motif analyses due to their limited number of cells and the scarcity of b-cCREs specific to this cell type (Fig. 3A).

We then applied two complementary analytical approaches to determine which sequence motifs are most important in distinguishing different classes of b-cCREs. First, we trained random forest classifiers using the HOCOMOCO collection of 401 TF-binding motifs (*64*) as features (see Materials and Methods). Motif significance is then defined as the average, normalized importance of an ensemble of random forest models. Second, we compared these results with a more classic sequence enrichment approach, the Analysis of Motif Enrichment (AME) tool from the MEME suite (*65*), applying the same HOCOMOCO catalog. For the b-cCREs active in each cell type, we plotted the feature importance from the random forest analysis against the enrichment value from AME for each motif. Results from both methods generally aligned, showing that motifs significantly enriched (AME score) within certain b-cCRE classes also had higher random forest feature importance (Fig. 3F, fig. S8, and table S5). Despite similarities in motifs, paralogous TFs display distinct expression profiles and tissue-specific regulatory roles. Moreover, TFs within the same structural family often share similar motifs. To discern the regulatory influences of specific TF proteins on b-cCREs across different brain cell types, we further integrated this motif analysis with TF expression levels from scRNA-seq data provided by PsychENCODE. We used the differential expression of TF genes in each cell type versus in all other cell types to identify the TFs that could correspond to the enriched motifs (Fig. 3G, fig. S9, and table S5).

In each cell type, several differentially expressed TFs show both high random forest feature importance and AME enrichment for their sequence motifs, and many of these TFs have documented activity specific to that cell type in the literature. Enriched motifs in excitatory neuron-specific b-cCREs include the neuronal differentiation family (NEUROD), early growth response factor (EGR), and the myocyte enhancer factor family (MEF) (Fig. 3F and table S5A), each of which has known roles in regulating development of neurons (*66*–*68*). scRNA-seq confirms that many TFs with enriched motifs are also differentially expressed in excitatory neurons (Fig. 3G and table S5B). TFs of NEUROD and EGR are expressed in significantly higher levels in excitatory neurons than other cell types (Wilcoxon rank sum test *P* < 2.22 × 10^−308^). MEF2A, MEF2B, MEF2C, and MEF2D motifs were all predicted as important, likely due to the high similarity among these motifs. However, scRNA-seq data suggest that only MEF2C is highly expressed in excitatory neurons compared with other cell types (Wilcoxon rank sum test *P* < 2.22 × 10^−308^) (Fig. 3G and table S5B). Similarly, we identify activator protein-1 (AP-1; heterodimers of JUN and FOS families) motif enrichment within inhibitory neuron-specific b-cCREs, as well as differential gene expression (FOSB in fig. S9 top-middle panel; table S5, C and D). TFs in the AP-1 family are involved in several processes related to neuronal development and function, including neuronal plasticity, memory formation, and neuronal regeneration (*69*). AP-1 TFs have been shown to regulate genes, such as brain-derived neurotrophic factor, that are essential for learning and memory (*70*, *71*). They are also essential for axonal regeneration, with c-JUN–deficient neurons causing neuronal atrophy and defective activation of glial cells (*72*).

Microglia-specific b-cCREs are enriched for a very different set of TFs, including members of the interferon regulatory factor (such as IRF8) and ELF/ELK/ETS (including SPI1 and SPIB) families (fig. S8, top right, and table S5E). IRF and SPI factors have known roles in immune cell development and function (*73*–*75*), and deep learning models of the immune system have identified TFs from the SPI and IRF families as potential developmental regulators, particularly in myeloid lineages, to which microglia belong (*76*). Integration with scRNA-seq data suggests that activity of *SPI1* (which encodes the PU.1 TF protein) and IRF8 is driving the regulatory activity in microglia (fig. S9, top right, and table S5F). Microgliogenesis requires both IRF8 and PU.1 (*77*). They act cooperatively to activate microglia by binding to a composite sequence motif; furthermore, they increase microglial activation under neurodegeneration conditions (*78*).

Integrating motif enrichment with TF gene expression data, we identified key TFs for different glial cell types: NFIA for astrocytes (fig. S9, top left, and table S5, G and H), SOX2 and SOX8 for oligodendrocytes (fig. S9, bottom left, and table S5, I and J), and OLIG2 and ASCL1 for OPCs (fig. S9, bottom middle, and table S5, K and L). These TFs have well-established functions for gliogenesis. During central nervous system development, neurons and glial cells are produced in a well-defined order (first neurons, then oligodendrocytes, and then astrocytes), and a key step in the switch of neural progenitors to gliogenesis is the induction of NFIA and Sox9, two proteins that promote the onset of gliogenesis and the arrest of neurogenesis (*79*). NFIA is a CCAAT box element-binding TF in the nuclear factor-1 (NFI) family whose gliogenic activity is required for the precise timing and levels of astrogenesis; inhibiting Nfia in the chick spinal cord leads to reduced glial precursor generation and premature neuronal differentiation, while its overexpression causes early astrocyte precursor migration (*80*). NFIA and NFIB are subsequently necessary for terminal astrocyte differentiation (*81*). Sox8 and Sox10, which belong to the HMG-box TF family, are specifically expressed in OPC and regulate their differentiation (*82*). Sox2 is essential for oligodendroglial proliferation and differentiation for the myelination and remyelination of postnatal mouse brain (*83*). Olig2, a patterning protein in the basic helix-loop-helix family, is essential for generating oligodendrocyte precursors and inhibiting ectopic astrocyte production in the mouse spinal cord (*84*). Expression of *Olig2* can induce oligodendrocyte precursors at ectopic locations (*85*). ASCL1 (also known as MASH1, in the basic helix-loop-helix family) is a proneural protein involved in specifying a subset of OPCs. *Olig2* and *Ascl1* remain expressed in OPCs, regulating their differentiation into myelinated oligodendrocytes (*86*).

### Deep neural networks predict chromatin accessibility at subsets of b-cCREs

The aforementioned autoencoder framework offers valuable insights into the sequence syntax of b-cCREs. An important caveat, however, is that it can only recognize that motifs are present within b-cCREs and not whether they function in a specific cell type. In other words, an autoencoder framework cannot determine which of the enriched motifs are bound by TFs or which motifs influence chromatin accessibility at a b-cCRE in a given cell type. To learn the relative functional importance of individual motifs in different cellular contexts, we trained additional models using the ChromBPNet framework (*87*). ChromBPNet is a convolutional neural network which learns to predict base pair–resolution chromatin accessibility profiles. It thus directly encodes the importance of individual motif sites in influencing chromatin accessibility and can also learn to impute footprints around TF binding sites to give insight into whether they are occupied.

We trained ChromBPNet on four sets of bulk ATAC-seq data from PsychENCODE: pooled ATAC-seq from FAN-sorted NeuN^+^ cells (neurons) in ventrolateral prefrontal cortex (VLPFC) and FAN-sorted NeuN^−^ cells (glia) in VLPFC (table S1). Last, we trained models for six different single cell types in the adult brain: excitatory neurons, inhibitory neurons, oligodendrocytes, oligodendrocyte precursors, astrocytes, and microglia, based on scATAC-seq data from Corces *et al.* (*19*). Each model passed the quality control standards recommended by ChromBPNet (table S6).

**Fig. 4.**
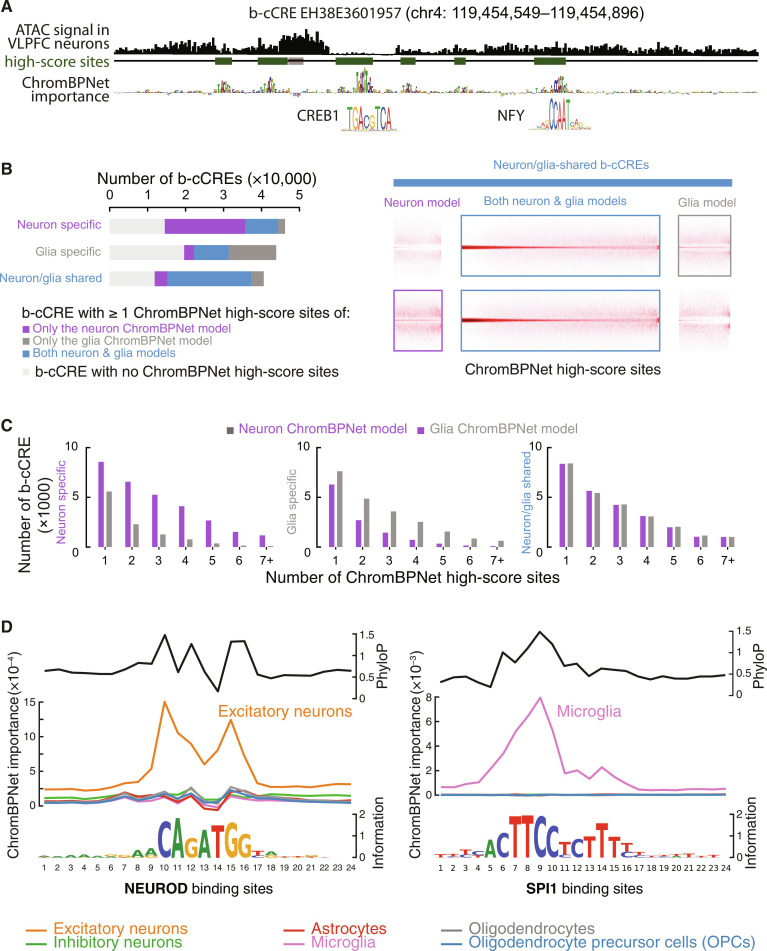
Deep learning of sequences influencing differences in chromatin accessibility at b-cCREs between neurons and glia. (**A**) Illustration of ChromBPNet-identified high-importance sequences within an example b-cCRE. Top track: VLPFC neuron ATAC-seq signal at the b-cCRE; middle track: regions within the b-cCRE which are high-importance score sites (green); bottom track: the reference human genome sequence scaled according to ChromBPNet profile importance scores. Motifs (CREB1 and NFY) matching high-importance regions are shown below the tracks. (**B**) Left: Number of neuron (top), glia (middle), or neuron/glia-shared b-cCREs (bottom) overlapping at least one high-importance score site from ChromBPNet models—neuron model (purple), glia model (gray), or both neuron and glia models (blue). b-cCREs that do not contain any called high-score sites are in light gray. Right: Heatmaps of ChromBPNet importance scores in 300-bp windows centered on high-score sites within neuron/glia-shared b-cCREs. Top row heatmaps show the glia model importance scores, and bottom row heatmaps show neuron model importance scores. Left column sites are called high-score only by the neuron model, center column sites are called high-score by both neuron and glia models, and right column sites are called high-score only by the glia model. (**C**) Histograms of b-cCREs according to the number of high-score sites (cTFBSs) from the neuron model (purple bars) or glia model (gray bars) they contain. Left plot shows neuron-specific, middle shows glia-specific, and right shows neuron/glia-shared b-cCREs. (**D**) Average ChromBPNet importance scores of de novo–discovered NEUROD (left) and SPI1 (right) binding sites from ChromBPNet models trained using the pseudo-bulk scATAC-seq signal profile in each cell type (colored accordingly), along with the average phyloP scores at those binding sites.

For each model, we generated base pair–resolution importance scores for all positions within b-cCREs using the DeepLIFT/DeepSHAP feature attribution method built into ChromBpNet (*88*, *89*). Positions which ChromBPNet predicts increased chromatin accessibility at a b-cCRE in a given cell type are assigned positive importance scores, while positions that ChromBPNet predicts decreased chromatin accessibility are assigned negative scores. All other positions are assigned scores near zero. In addition to importance scores, we used ChromBPNet to impute base pair–resolution pseudo-bulk scATAC-signal profiles at each b-cCRE for each cell type. In some cases, these imputed signal profiles have higher resolution than the underlying scATAC-seq data and can highlight footprints where bound TFs are predicted to protect a motif site from the insertion of the Tn5 transposase in the scATAC-seq experiments. To highlight these features, we illustrate importance scores and imputed signal profiles computed by ChromBPNet at the b-cCRE with the highest NeuN^+^ ATAC-seq signal (Fig. 4A). Visually, short stretches of high-scoring nucleotides resemble known TF binding motifs: This b-cCRE exhibits five copies of CREB1 binding sites at various importance scores and a high-scoring NFY binding site (Fig. 4A).

Notably, the ChromBPNet models have no knowledge of b-cCRE labels, such as whether a b-cCRE is neuron-specific, glia-specific, fetal-specific, etc. Thus, these labels offer a valuable way to validate our models. For example, we can ask whether the VLPFC neuron ChromBPNet model is more likely than the VLPFC glia ChromBPNet model to identify important sequences within neuron-specific b-cCREs. To do this, we first needed to design an approach to identify the boundaries of high-scoring sites [candidate TF binding sites (cTFBSs)] based on ChromBPNet importance scores. We trained a hidden Markov model (HMM) to recognize subsequences within b-cCREs that have a higher density of high-scoring or low-scoring positions (see Materials and Methods). We find that HMM-identified cTFBSs identified by the neuron model but not the glia model are more prevalent in neuron-specific b-cCREs, while cTFBSs from the glia model are more prevalent in glia-specific b-cCREs (Fig. 4B, left). HMM-identified cTFBSs present in neuron/glia-shared b-cCREs tend to be identified as important by both neuron and glia ChromBPNet models (Fig. 4B, left). Similarly, individual neuron-specific b-cCREs contain a larger number of HMM-identified cTFBSs from the neuron models, while glia-specific b-cCREs contain a larger number of cTFBSs from the glia models (Fig. 4C).

### Neuron/glia-shared b-cCREs use different sequence syntax in different cell types

Although most of the HMM-identified cTFBSs in Fig. 4B at neuron/glia-shared b-cCREs are identified by both the neuron and glia ChromBPNet models, some are specific to one cellular context or the other. We sorted these motifs according to their importance in each cell type and found that many are assigned positive importance scores by ChromBPNet only in neurons and some only in glia (Fig. 4B, right). In contrast, cTFBSs identified by both models have consistently high-importance scores in both the neuron and glial models (Fig. 4B, right). This implies that chromatin accessibility at some neuron/glia-shared b-cCREs is modulated by different TFs in different contexts.

Combining the importance scores from ChromBPNet and deep-learned TF motifs from cell type–specific b-cCREs, we are able to highlight differences in predicted TF activity in individual brain cell types (Fig. 4D and fig. S10). *NEUROD*, for example, plays a critical role in neuronal differentiation (*66*). Examining deep-learned NEUROD binding sites in excitatory neuron-specific b-cCREs from the Corces *et al.* (*19*) single-cell dataset reveals lineage-specific differences in ChromBPNet importance scores, with excitatory and inhibitory neurons showing the highest average importance across all examined NEUROD binding sites (Fig. 4D). Similarly, SPI1 is known for its role in myeloid cell development (*73*). Deep-learned SPI1 binding sites are only important in the microglia ChromBPNet model (Fig. 4D). Binding sites of the SOX family of TFs show the highest ChromBPNet importance in oligodendrocytes, followed by astrocytes and OPCs (fig. S10A), consistent with the importance of SOX factors in the oligodendrocyte lineage (*90*). In comparison, the NFIA binding sites show the highest ChromBPNet importance in astrocytes, followed by OPCs and oligodendrocytes (fig. S10B), consistent with its importance for gliogenesis in general and specifically in the astrocyte lineage (*79*, *81*). AP-1 binding sites show high ChromBPNet importance in neurons, with inhibitory neurons higher than excitatory neurons (fig. S10C), consistent with its function in neuronal plasticity, memory formation, and neuronal regeneration (*69*). IRF sites exhibit high ChromBPNet importance only in microglia (fig. S10D), and IRF8 is known to play essential roles in microglial activation and migration (*77*).

### Neuron-specific and fetal-specific b-cCREs are most conserved across mammals, while other classes are actively evolving

We explored in more detail the evolutionary trajectory of various classes of b-cCREs throughout the mammalian and primate lineages. As described above, b-cCREs are more conserved than nb-cCREs in both mammals and primates (Fig. 1A and fig. S1, E to H). We asked whether different subsets of fetal versus adult or neuron versus glia b-cCREs display different patterns of evolutionary conservation than other subsets.

**Fig. 5.**
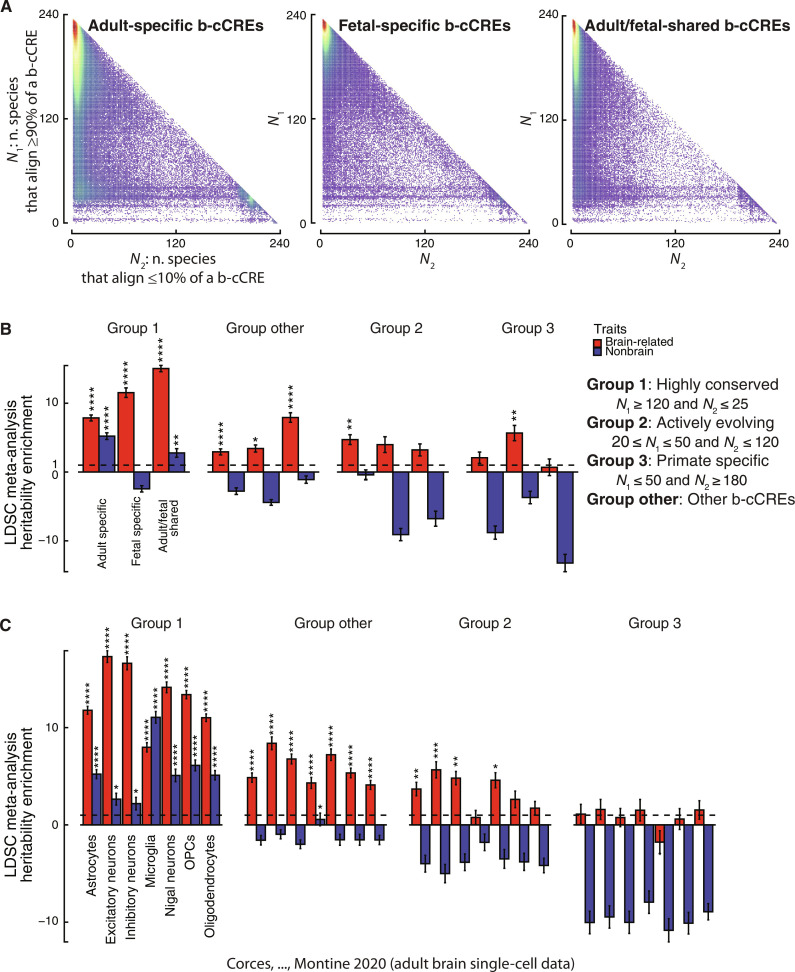
Evolutionary conservation of b-cCREs throughout the mammalian lineage and the role of conserved versus evolving elements in complex traits. (**A**) Evolutionary conservation of b-cCREs according to the number of mammalian genomes in which they align. *N*_1_ indicates the number of genomes aligning ≥90% of the b-cCRE’s sequence. *N*_2_ denotes the number of genomes aligning ≤10% of the b-cCRE’s sequence. From left to right, only adult-specific, fetal-specific, and adult/fetal-shared b-cCREs are shown. (**B**) Heritability enrichment meta-analysis of adult-specific, fetal-specific, and adult/fetal-shared b-cCREs, split by evolutionary groups, in brain-related traits (red) and nonbrain-related traits (blue). LDSC meta-analysis *P* value for enrichment in heritability of genetic variants residing in subsets of b-cCREs: *P < 0.05, **P < 0.01, and ****P < 0.0001. (**C**) Heritability enrichment meta-analysis of single-cell–type–specific b-cCREs, split by evolutionary groups, in brain-related traits (red) and nonbrain-related traits (blue). LDSC meta-analysis *P* value for enrichment in heritability of genetic variants residing in subsets of b-cCREs: *P < 0.05, **P < 0.01, ***P < 0.001, and ****P < 0.0001.

We first leveraged the multiple-genome alignments of 240 mammals from the Zoonomia Consortium (*37*) to examine fetal- and adult-specific b-cCREs and adult/fetal-shared b-cCREs. For each b-cCRE, we asked how many of the mammalian genomes align more than 90% and how many align less than 10% of the b-cCRE sequence (*38*). When plotted on the *x*-*y* plane according to these values, the b-cCREs form a triangle given the two categories are mutually exclusive for a given species (Fig. 5A and fig. S11, A to C). Fetal-specific b-cCREs exhibit greater density in the upper left corner than the other categories, indicating that they align at >90% of their sequence in more mammals and are thus more conserved on average than adult and shared b-cCREs, which, in contrast, form a vertical stripe on the left edge of the triangle (Fig. 5A). Elements in this stripe align at least 10% of their sequence in most mammals, indicating that they are not brand new primate- or human-specific elements. However, they align >90% of their sequence in very few mammals, suggesting that they are actively evolving through local sequence mutations.

Next, we compared the evolutionary conservation of neuron-specific b-cCREs and glia-specific b-cCREs using the same method. Neuron-specific b-cCREs occupy primarily the upper left corner of the mammalian alignment triangle (fig. S11B), indicating greater overall evolutionary conservation than glia-specific and neuron/glia-shared b-cCREs. In contrast, both glia-specific and neuron/glia-shared b-cCREs form the same vertical stripe as adult-specific and adult/fetal-shared b-cCREs, indicating active evolution in mammals via small local sequence mutations (compare fig. S11B with fig. S1, G and H) and greater overall evolutionary conservation than glia-specific and neuron/glia-shared b-cCREs. This analysis suggests that a greater fraction of the cis-regulatory information used by glia in the adult brain has undergone recent sequence evolution in primates compared with cis-regulatory information used by adult neurons. Human brains display more diverse and structurally complex astrocytes than other mammalian brains, and grafting of human astrocytes into mouse brains enhances learning and memory (*91*, *92*); meanwhile, radial glia appear to be involved in cortical expansion and folding (*93*). Therefore, our results are consistent with previous work showing that genes near human-accelerated regions are strongly expressed in astrocytes and radial glia (*94*) and may lend insight into the evolution of higher-order cognitive functions.

### Primate conservation is more informative than mammalian conservation in identifying trait-associated b-cCREs

Mammal- and primate-constrained positions in the human genome identified by the the Zoonomia Consortium are strongly enriched for heritability for complex traits (*39*). Similarly, we previously showed that cis-regulatory elements conserved across mammals based on Zoonomia annotations are the strongest contributors to heritability for a panel of 69 distinct complex traits and diseases (*38*). A few of these traits are brain-related, but most are not. We therefore asked whether the most evolutionarily constrained b-cCREs are the strongest contributors to heritability for brain-related traits, or whether nonconstrained b-cCREs play a role. To this end, we performed LDSC meta-analysis on a variety of subsets of b-cCREs stratified by evolutionary conservation.

First, we divided fetal-specific, adult-specific, and adult/fetal-shared b-cCREs into three groups of conservation based on the mammalian-genome-alignment triangle described above as we did previously in our Zoonomia analysis (*38*). Group 1 (G1) b-cCREs align >90% in a majority of mammals (strongly conserved), group 2 (G2) b-cCREs align >90% in some mammals and <10% in others (actively evolving), while group 3 (G3) b-cCREs align <10% in most mammals except primates (primate- or human-specific) (see Materials and Methods for details). LDSC revealed enrichment for brain-related traits in adult-specific, fetal-specific, and adult/fetal-shared G1 b-cCREs (7.86-, 11.54-, and 15.04-fold; enrichment *P* = 4.45 × 10^−15^, 8.32 × 10^−27^, and 2.54 × 10^−33^, respectively; Fig. 5B). We also observed enrichment for nonbrain-related traits for adult-specific G1 b-cCREs (5.22-fold, enrichment *P* = 6.90 × 10^−10^; Fig. 5B), albeit significantly less than for brain-related traits (*z* score *P* = 4.78 × 10^−3^ for difference). This enrichment derives predominantly from immune-mediated traits such as blood cell characteristics and autoimmune diseases (table S4K); we hypothesize that it results from pleiotropic effects at strongly conserved regulatory elements active in other cell types beyond brain. Last, we observed enrichment for brain-related traits (neruroticism and worry) for fetal-specific G3 b-cCREs (5.67-fold; enrichment *P* = 1.04 × 10^−3^), suggesting that some recent primate innovations in regulatory sequence involved in fetal brain development contribute to complex brain-related traits. In contrast, adult-specific or adult/fetal-shared G3 b-cCRE groups did not display significant enrichment (Fig. 5B).

To obtain a finer-grained view of the relative conservation of trait-associated regulatory elements, we divided the conservation triangle from Fig. 5A into 16 slices with roughly equal numbers of cCREs, derived from four rows and columns based on the relative density of cCREs in the triangle (fig. S11C; see Materials and Methods). This enables us to determine at higher resolution whether actively evolving regulatory elements contribute to trait heritability. We performed this analysis both for the adult-fetal axis and subsets of b-cCREs defined based on the scATAC-seq signal. In agreement with the previous results, b-cCREs in the upper left triangle (slices 12 to 14) were enriched for brain-specific traits for every b-cCRE subset we tested (compare fig. S11D with Fig. 5B). Notably, we also observe enrichment for most b-cCRE subsets within the vertical, actively evolving stripe highlighted in Fig. 5A, corresponding to slices 4, 5, and 8 to 10 (fig. S11D), indicating that b-cCREs that evolved early in mammalian evolution but continue to undergo active sequence turnover contribute to brain-related traits as well. In addition, this analysis highlights that the G3 fetal-specific b-cCRE enrichment for brain-related traits (Fig. 5B) derives predominantly from partitions of regulatory elements conserved across primates but not present in other mammals (slices 2 and 7 in fig. S11D).

Similarly, we examined trait heritability enrichment for regulatory elements active in various brain cell types based on single-cell data stratified by evolutionary conservation groups (Fig. 5C). G1 b-cCREs displayed strong heritability enrichment across the traits for all single-cell types, with brain traits displaying greater enrichment than nonbrain traits for all cell types except microglia. For G2 b-cCREs and b-cCREs not classified as G1, G2, or G3 (group other), brain traits displayed significant enrichment, while nonbrain traits did not. No enrichment was observed for G3 b-cCREs active in single-cell type, largely due to their small partition sizes (Fig. 5C). We further analyzed the 16 slices of the conservation triangle for single-cell data and observed enrichment for the most conserved b-cCREs (upper triangle, slices 12 to 15) and the actively evolving b-cCREs (vertical stripe, slices 4, 5, and 8 to 10) in all brain single-cell types for brain-related traits (fig. S12). The enrichment of the primate-specific b-cCREs for brain-related traits derive primarily from excitatory neurons (slices 2 and 7 in fig. S12).

### Primate-conserved positions in b-cCREs play a large role in brain-related trait heritability complementing pan-mammalian conserved positions

**Fig. 6.**
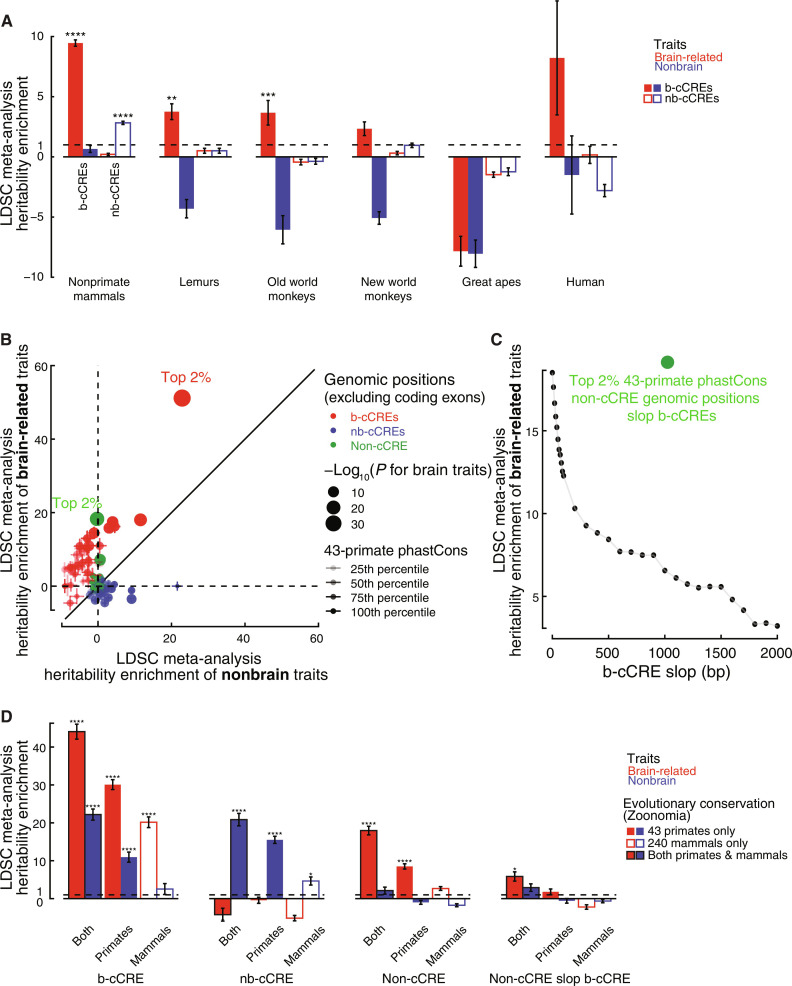
Identifying primate-conserved b-cCRE sequence elements and characterizing their role in complex traits. (**A**) Heritability enrichment meta-analysis of b-cCREs and nb-cCREs, split based on primate conservation, in brain-related traits (red) and nonbrain-related traits (blue). LDSC meta-analysis *P* value for enrichment in heritability of genetic variants residing in subsets of b-cCREs: **P < 0.01, ***P < 0.001, and ****P < 0.0001. (**B**) Comparison of heritability enrichment in brain-related traits (*x* axis) and nonbrain-related traits (*y* axis) cCREs, divided into 50 bins based on primate conservation and then further divided by b-cCRE (red), nb–cCRE (blue), and non-cCRE (green). (**C**) Heritability enrichment meta-analysis of brain-related traits (*y* axis) of the top 2% of primate constrained positions not intersecting a cCRE after removing those within *x* bp of a b-cCRE (b-cCRE slop). (**D**) Heritability enrichment of mammalian-constrained positions, primate-constrained positions, or both, intersecting b-cCREs, nb-cCREs, non-cCREs, and non-cCRE slop b-cCRE in brain-related traits (red) and nonbrain-related traits (blue). LDSC meta-analysis *P* value for enrichment in heritability of genetic variants residing in subsets of b-cCREs: *P < 0.05 and ****P < 0.0001.

Motivated by our results on the enrichment of primate-specific b-cCREs in brain-related traits, we asked whether primate-conserved positions or mammalian-conserved positions within b-cCREs and nb-cCREs are more or less enriched for brain- and nonbrain-related trait heritability. To answer this question, we first stratified b-cCREs according to their estimated evolutionary origin based on the most distant species from humans in which the b-cCRE aligns (see Materials and Methods). b-cCREs predating the split of primates from other mammals display the strongest heritability enrichment for brain-related traits, while nb-cCREs predating the primate split display the strongest heritability for nonbrain-related traits (9.46-fold; enrichment *P* = 1.38 × 10^−49^ and 2.83-fold; enrichment *P* = 6.63 × 10^−36^, respectively, Fig. 6A and table S4L). b-cCREs shared with species as distant as lemurs and b-cCREs shared with species as distant as old-world monkeys both display enrichment for brain-related traits as well (3.75-fold; enrichment *P* = 1.62 × 10^−3^ and 3.67-fold; enrichment *P* = 7.10 × 10^−4^, respectively), while nb-cCREs at such evolutionary distances are no longer enriched for nonbrain traits (Fig. 6A). Groups of b-cCREs having evolved more recently do not show significant enrichment for brain-related traits, although we do note that human-specific b-cCREs which arose after the split of humans from chimpanzees show sevenfold heritability enrichment for brain traits, albeit with a large standard error owing to this group’s small number (Fig. 6A).

Next, we asked whether highly conserved positions within elements contribute to trait heritability more than less-conserved positions. To answer this question, we binned all genomic positions according to their rank based on phastCons score across 43 primates from Zoonomia (see Materials and Methods). We further divided each bin into positions intersecting b-cCREs, positions intersecting nb-cCREs, or positions outside of cCREs. We ran this analysis twice, once with exonic positions included and once with exonic positions excluded; the results were nearly identical because most GWAS-identified variants are noncoding, so only the results excluding coding exons are presented. Overall, the top 2% of primate-conserved positions within b-cCREs display by far the strongest enrichment of any bin for brain-related traits (51.16-fold, enrichment *P* = 4.80 × 10^−37^), as well as a substantially lower enrichment for nonbrain-related traits (22.90-fold, enrichment *P* = 1.94 × 10^−29^). Primate-conserved positions in nb-cCREs are, in contrast, enriched for nonbrain-related traits and not brain-related traits (Fig. 6B). We also observed that primate-conserved positions outside of b-cCREs and cCREs exhibit high heritability enrichment for brain-related traits (18.29-fold, enrichment *P* = 1.67 × 10^−22^; Fig. 6B). To investigate the source of this enrichment, we first excluded genomic positions flanking coding exons but observed little effect, indicating that the signal does not reside in splice sites. We then iteratively excluded conserved positions within expanding windows around b-cCREs and reran this analysis; the enrichment turns out to derive predominantly from conserved positions no more than 500-bp away from the nearest b-cCRE (Fig. 6C). Genomic positions flanking but not within regulatory elements can still modulate their chromatin accessibility and regulatory functions; thus, we concluded that such positions near b-cCREs are the source of the heritability enrichment in the non-cCRE partition of the genome.

Last, we used our b-cCRE annotations to refine analysis from the Zoonomia Consortium, which demonstrated that highly primate-conserved positions exhibit greater heritability enrichment for complex traits than mammal-conserved positions in the human genome (*38*). Zoonomia defined two sets of roughly 100 million each, highly conserved positions in the human genome: those in the top 1% most conserved across 43 primates (highest 43-primate phastCons scores) and those across 240 mammals (highest 240-mammal phyloP scores). Intersection of these two sets yields 54.4 M primate-only, 53.9 M mammal-only, and 46.7 M primate and mammal conserved positions in the human genome, and the first and third subsets displayed greater trait heritability than the second for the 69 traits considered by Zoonomia (*38*). We further stratified positions in each of these partitions according to whether or not they intersect a b-cCRE, nb-cCRE, or no cCRE. Consistent with the results of Zoonomia, b-cCRE positions in the top 1% by both mammalian phyloP and primate phastCons were most enriched for brain-related traits, followed by b-cCRE positions highly conserved across primates alone and then those highly conserved across all mammals (44.06-, 30.08-, and 20.15-fold; enrichment *P* = 1.75 × 10^−29^, 1.04 × 10^−25^, and 5.45 × 10^−14^, respectively; Fig. 6D, left). For nb-cCREs, the conservation pattern was the same, but only nonbrain-related traits enriched (Fig. 6D, left middle). Similarly to the above result (Fig. 6B), primate-conserved positions and primate/mammal-conserved positions outside of cCREs are enriched for brain-related traits but not nonbrain traits (Fig. 6D, right middle), and this enrichment was all but eliminated when positions in close proximity to b-cCREs but not within them were excluded (Fig. 6D, right), consistent with our earlier analysis on the top 2% genomic positions with the highest primate conservation (Fig. 6C). Together, these results highlight that b-cCREs and their flanking regions conserved across primates play a key role in a variety of behavioral and psychiatric traits.

### PsychSCREEN offers interactive visualization of brain regulatory elements and associated annotations

To make the resources described in this work accessible to the broader research community, we designed and implemented PsychSCREEN, a web-based platform for data search and visualization (https://psychscreen.wenglab.org/). PsychSCREEN provides four distinct portals which offer interactive visualization of the genomic annotations described here from different biological perspectives, alongside some of the PsychENCODE data and public datasets used to derive those annotations. The download page offers all the b-cCRE subsets, cTFBSs, computational models, and GWAS analyses described here in common bioinformatic data formats. To facilitate interactive visualization, PsychSCREEN uses the ReactJS framework, features a minimalistic design with salient Portal illustrations for intuitive navigation, and includes an embedded genome browser which renders visualizations in scalable vector graphics (SVG), which can themselves be exported to aid users in generating figures and sharing biological insights. The architecture of PsychSCREEN is described in detail in Materials and Methods.

**Fig. 7.**
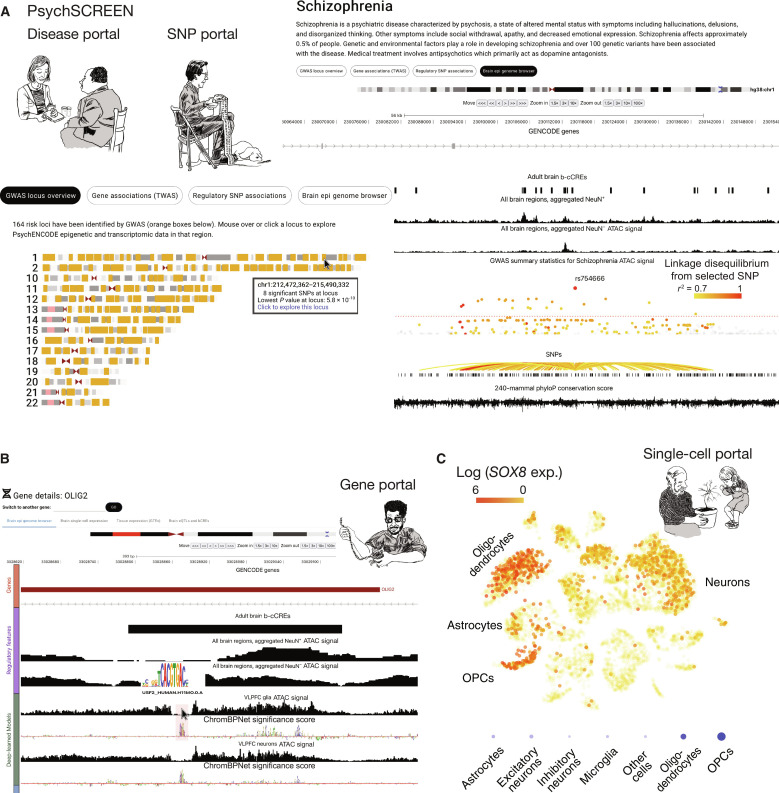
PsychSCREEN is an interactive platform to view and compare multiomic data and annotations. (**A**) Screenshots from PsychSCREEN’s disease/SNP portals, including a written description of the queried disease—schizophrenia (top right)—and a view of all risk loci identified by GWAS and where they appear in the genome (bottom left). Clicking on a locus redirects you to a genome browser (bottom right) spanning the highlighted coordinates, displaying tracks representing b-cCREs, aggregate neuron and glia ATAC signal, Schizophrenia GWAS summary statistics, a set of documented SNPs and their linkage disequilibrium, and base pair–resolution mammalian conservation scores. (**B**) Screenshots from PsychSCREEN’s gene portal, upon searching for the gene *OLIG2*. The genome browser is the default view, displaying b-cCREs, aggregate neuron and glia ATAC signal, and ATAC signal on an individual experiment and predicted importance scores from ChromBPNet models of PsychENCODE data. On the ChromBPNet track, highlighting a section of nucleotides (shaded in red) will prompt a search of the closest matching TF motif, and the motif logo is displayed. (**C**) Screenshots from PsychSCREEN’s single-cell portal, upon searching for the gene *SOX8*. The UMAP plot from a scRNA-seq experiment is displayed (top), with the color of each dot representing the expression (natural log-transformed counts per 10^5^ total sequencing reads) of the queried gene. Alternatively, a dotplot (bottom) can be displayed, with the size of the circle representing the percentage of cells in a particular cluster expressing the queried gene and the opacity representing the average expression of the gene in that cluster.

PsychSCREEN’s Disease and Trait portal provides access to the GWAS analyses described here for brain-specific traits. The user searches for a disease or trait of interest, such as schizophrenia or neuroticism. The portal uses summary statistics for the trait to identify risk loci, which it plots on cytoband views of chromosomes. Highlighted loci can be clicked, which leads the user to an interactive genome browser view which displays significant single nucleotide polymorphisms (SNPs) in a Manhattan-style plot at the locus alongside annotations, including a combined set of adult and fetal b-cCREs, ChromBPNet importance scores, and pseudo-bulk tracks from single-cell types (Fig. 7A). A related *SNP/QTL* Portal displays PsychENCODE and PsychSCREEN annotations, including expression quantitative trait loci (eQTLs), b-cCRE intersections, cTFBS intersections, and trait associations for a searched SNP, and allows the user to visualize these annotations in the SNP’s neighborhood using an embedded genome browser view. Other features of the Disease and Trait portal include a transcriptome-wide association study (TWAS) tab which displays TWAS results from PsychENCODE if available for a given trait and a “regulatory SNP associations” tab which allows the user to export risk variants for the trait which intersect b-cCREs and cTFBSs for downstream analyses.

PsychSCREEN’s gene portal provides access to gene regulatory annotations for individual genes of interest (Fig. 7B). The user inputs the name of a gene and is directed to a genome browser view which displays the genomic neighborhood around the gene, with overlays showing nearby b-cCREs, importance scores from ChromBPNet models, and cTFBSs. The user can also toggle tracks from single-cell and bulk ATAC-seq datasets from PsychENCODE. In addition, the gene portal offers gene expression tabs, which display expression of the gene in the brain across adult and fetal time points based on PsychENCODE data and its expression in various tissues and brain regions based on Genotype-Tissue Expression (GTEx) data (*95*). An eQTL and b-cCRE tab allows the user to view SNPs correlated with expression of the given gene based on annotations from PsychENCODE studies. In addition, a single-cell expression tab displays expression patterns of the gene in individual brain cell types based on scRNA-seq data of the adult dorsolateral prefrontal cortex produced by PsychENCODE. Last, a single-cell portal offers interactive exploration of PsychENCODE single-cell datasets, including scRNA-seq differential gene expression, scATAC-seq peaks, single-cell QTLs (i.e., SNPs and associated genes), and cell type–specific gene regulatory networks, including links between enhancers and promoters (Fig. 7C).

Users interested in programmatic access can use an associated GraphQL application programming interface (API) to query the datasets included in PsychSCREEN. This API is publicly available at https://psychscreen.api.wenglab.org/graphql and is described in more detail in Materials and Methods.

## DISCUSSION

We have integrated chromatin accessibility and transcriptomic data from ENCODE and PsychENCODE to construct an atlas of b-cCREs. Numbering 361,844 elements, these represent a substantial expansion of previous catalogs and, to our knowledge, one of the most comprehensive collections of brain-active regulatory information available to date. Analyzing fetal and adult datasets, we find that roughly one-third of these b-cCREs are specific to the first- and second-trimester fetal brain, and one-third are specific to the adult brain. On the basis of data from FAN-sorted adult neurons and glia, we further classify approximately 40,000 adult b-cCREs as neuron-specific and another 40,000 as glia-specific.

Our list of b-cCREs covers approximately 95% of the ATAC-seq peaks obtained from scATAC-seq data. This represents a much higher coverage than the bulk ATAC-seq data derived from the same biosamples in the original study, which only covered 34% of the scATAC-seq peaks (*19*). Anchoring regulatory elements on b-cCREs offers several additional advantages. First, b-cCREs have well-defined boundaries and are similar in size to nucleosomes. They can serve as reference elements for scoring pseudo-bulk ATAC-seq signals, facilitating straightforward comparisons between different cell types within the same scATAC-seq dataset, as well as between different ATAC-seq datasets. Second, as b-cCREs are subsets of cell type–specific cCREs, they are accessioned and associated with the extensive data and annotations generated by the ENCODE project and other studies on ENCODE samples.

Past analyses of GWAS have clearly demonstrated that heritability for behavioral and psychiatric traits is enriched in genomic regions which are gene regulatory in the human brain (*17*–*19*, *53*). The extent to which these effects are neurodevelopmental versus active in the adult brain is an area of active investigation, and likewise the extent to which the effects are neuron-specific, glia-specific, or shared between neurons and glia. The importance of adult versus fetal and neuronal versus glial effects varies by trait, with microglial gene dysregulation playing a key role in Alzheimer’s disease (*96*–*98*), for example, and neuronal gene dysregulation being involved in schizophrenia (*99*–*102*). We find heritability enrichment for a variety of behavioral and psychiatric traits within neuronal and glial regulatory elements in both the adult and fetal brain. On the whole, both adult and fetal regulatory elements are important for these traits by our analysis, but neurodevelopmental elements are more enriched. Similarly, while both neuronal and glial elements are important by our analysis, neuronal elements appear to play a significantly greater role in these traits.

To gain an understanding of how these variants influence the development of traits and diseases, it is essential to model and understand the molecular function of regulatory elements. Machine learning approaches, particularly deep neural networks, are powerful tools for predicting how sequence influences TF binding and chromatin accessibility (*103*). These models can be used to prioritize variants that are most likely to be causal for psychiatric traits. For example, recent work modeling chromatin accessibility in the human frontal cortex with neural networks predicts that de novo mutations may cause autism spectrum disorder by affecting TF binding in cortical neurons (*104*). Extending these approaches to our expanded set of b-cCREs, we identify hundreds of thousands of cTFBSs, which we predict modulate chromatin accessibility in neurons and glia. We show that heritability for brain-related traits is particularly enriched within these sites, supporting their likely functional roles.

In addition to prioritizing risk variants, analysis of these sites provides insight into TFs which are most important in driving neurodevelopment and the differentiation of brain-constituent cell types. In support of earlier studies, we found motifs of distinct families of TFs for neurons (NEUROD, EGR, MEF, and AP-1) (*66*–*68*), astrocytes (NFIA) (*81*), microglia (IRF and SPI1) (*73*–*75*), oligodendrocytes (SOX) (*105*), and oligodendrocyte precursor cells (OLIG) (*66*, *106*) in b-cCREs that are likely to function in each cell type based on scATAC-seq data. These findings are based on motifs enriched in brain cell type–specific b-cCREs identified through scATAC-seq data. Our method of integrating motif enrichment with TF expression data at the single-cell level effectively pinpoints the specific TFs responsible for cell type–specific regulation. In addition, even for the b-cCREs that are open chromatin in all brain-related cell types, our analysis revealed the binding sites of distinct TFs in each cell type, indicating complex and cell type–specific regulatory syntax.

A limitation of our study is its reliance on chromatin accessibility as an indicator of potential functionality in brain cell types or developmental stages (fetal versus adult). While we compared our b-cCRE atlas with existing databases of regulatory elements in brain cells, our approach primarily involved computational analyses. These included examining single-cell gene expression, assessing evolutionary conservation in mammalian and primate lineages, and analyzing heritability enrichment of GWAS variants associated with brain-related and other traits. However, there is a pressing need for experimental validation of these b-cCREs within the relevant brain cell types. Such studies are crucial for a deeper understanding of b-cCREs’ roles in gene regulation and the mechanisms driving these functions.

Last, by integrating the latest analysis from the Zoonomia Consortium (*37*), we are able to gain unprecedented resolution for studying the evolution of brain-active cis-regulatory information throughout the mammalian lineage. Previous studies have demonstrated that brain-active regulatory elements are enriched within human-accelerated regions, which are sequences that evolved early in mammalian evolution but have undergone accelerated sequence turnover in humans specifically (*34*, *107*). These elements appear to regulate gene expression in glia somewhat more than in neurons (*94*), and indeed the physiology of human astrocytes (*91*, *92*) as well as radial glia–induced cortical folding may represent key features accounting for the unique cognitive abilities of the human brain (*93*). Our results support these findings: Our analysis suggests that smaller sequence mutations within mammalian-conserved elements are more common among b-cCREs than emergence of entirely new human- or primate-specific sequences, for example, by transposable element insertion. Furthermore, our results indicate several thousand primate-specific b-cCREs, in particular those that may function in fetal brains and excitatory neurons and astrocytes that show enrichment for brain-related human traits.

Overall, our work represents a comprehensive atlas of regulatory information in the human brain, along with computational models predicting how this information functions at a molecular level. Our analysis suggests that these elements and associated models will be of great value in studying the pathophysiology and evolution of brain-related traits. By presenting our results with a compilation of PsychENCODE single-cell data and public data, we aim to facilitate the understanding of brain functions at individual genes, regulatory elements, variants, and traits.

## MATERIALS AND METHODS

### Defining b-cCREs

We developed a consensus-based approach to define b-cCREs, using data from 96 DNase-seq experiments conducted on adult brain tissues and 14 DNase-seq experiments on fetal brain tissues, all generated by the ENCODE consortium (table S1). Our approach comprises two distinct phases.

In the initial phase, we predicted a list of cCREs that might exhibit activity in each respective biosample using the DNase-seq data in that specific biosample. Following the methodology established by the ENCODE consortium (*15*), we initiated the process by downloading bigWig files for the DNase-seq experiments, containing read-depth normalized DNase signal data. We then used the UCSC tool, bigWigAverageOverBed, to calculate the average signal across the genomic positions of each of the 2.9 M representative DNase hypersensitive sites (rDHSs), which encompass the 2.35 M cCREs (version 4 cCREs within the ENCODE project) and 0.55 M additional regions that facilitate data normalization. The DNase signals at the 2.9 M rDHSs were log-transformed and normalized as *z* scores, following a normal distribution. We retained the 2.35 M V4 cCREs for all subsequent analyses. cCREs with *z* scores exceeding 1.64 (equivalent to the 95th percentile for a one-tailed *Z* test) were classified as active cCREs within a specific experiment. This initial phase was carried out across all 96 adult and 14 fetal DNase-seq experiments, resulting in a distinct list of active cCREs for each experiment.

In the second phase of our approach, we arrived at a consensus regarding the active cCREs, defining adult b-cCREs as those 253,638 cCREs active in five or more of the 96 DNase experiments conducted on adult biosamples. Similarly, fetal b-cCREs were defined as all cCREs exhibiting activity in five or more fetal brain biosamples. Because fetal brain tissue is difficult to obtain and the ENCODE DNase-seq data on fetal brain samples are of particularly high quality, in the case of fetal b-cCREs, we also incorporated cCREs identified as active in one to four fetal biosamples, provided their *z* scores exceeded 2.32 (equivalent to the 99th percentile) in any fetal brain biosample. The combination of the two sources resulted in a total of 230,936 fetal b-cCREs. From these adult and fetal b-cCRE lists, we created a final list containing b-cCREs found in either the fetal or adult b-cCRE lists or both. The b-cCREs unique to the adult list or the fetal list are called adult-specific and fetal-specific b-cCREs, respectively, and the b-cCREs shared by the two lists are called adult/fetal-shared b-cCREs.

### Evaluating the coverage of b-cCREs using scATAC-seq data

We curated four scATAC-seq datasets to understand the cell type specificity and overall coverage of the b-cCRE set (table S1). FASTQ files for Corces *et al.* (*19*) were obtained from Gene Expression Omnibus (GEO) accession GSE147672. We used cellranger-atac to align reads to GRCh38 and used the cell type assignments in Corces *et al.* (*19*) to generate pseudo-bulk alignment files. These alignment files were then converted to bigWig using deepTools bamCoverage (*108*).

Fragment files from Domcke *et al.* (*20*) (for both brain and nonbrain biosamples) were downloaded directly from the web page (https://descartes.brotmanbaty.org/) accompanying their publication, along with cell barcode metadata. These fragment files were split according to published cell type, then lifted over to GRCh38, using UCSC liftover (*109*), and converted to bigWig using deepTools bamCoverage.

bigWigs from Morabito *et al.* (*54*) was downloaded from their UCSC genome browser, selecting only their healthy controls from each cell type (https://genome.ucsc.edu/s/smorabito/AD_snATAC). bigWigs from Zhang *et al.* (*53*) was provided by the authors directly.

For each pseudo-bulk bigWig file, we computed the signal across each cCRE using a custom Python script, followed by log transformation and *z* score normalization. Active cCREs were defined as those with a *z* score > 1.64 in a particular experiment. Data from Corces, Morabito, and Zhang (*19*, *53*, *54*) were processed in GRCh38. Any file or cell type that called less than 10,000 cCREs was removed from the analysis.

From a list of active cCREs for each individual cell type, we identified the 50,000 highest-scored cCREs. If a cell type had fewer than 50,000 active cCREs, then we just used however many cCREs were called. From these top 50,000 active cCREs, we computed the proportion of those that were also classified as b-cCREs.

### Computing NeuN^+^ and NeuN^−^ ATAC-seq signals and calling active b-cCREs in various brain regions

We obtained NeuN FAN-sorted ATAC-seq data from 14 different regions of adult brain (*6*, *52*) and processed them using the ENCODE uniform processing pipeline. For 63 biosamples, ATAC-seq data were generated for both NeuN^+^ and NeuN^−^ subsets of nuclei. For each of these, we used the fold change over control bigWigs to compute the log-transformed ATAC signal at cCREs, followed by *z* score normalization. Any b-cCRE with a *z* score greater than 1.64 was called as active in that particular ATAC-seq experiment. In fig. S5, these called b-cCREs were used to compute the overlap coefficient between biosamples in a pair-wise manner, comparing NeuN^+^ biosamples and NeuN^−^ biosamples separately. These results were then plotted using the pheatmap package in R.

We also used these bigWigs to generate aggregate signal tracks representing the average regulatory landscape of neurons and glia in the brain. First, for each of the 14 brain regions, all biosamples isolated from that region (NeuN^+^ and NeuN^−^ separately) were summed using UCSC’s bigWigMerge, followed by UCSC’s bedGraphToBigWig. These aggregate bigWigs were used to compute active b-cCREs from each brain region, which were then used to compute the overlap coefficient between brain regions and plot the results in Fig. 1F using the pheatmap package in R.

There were 63 NeuN^+^ (neuron) biosamples and 65 NeuN^−^ (glia) biosamples. The fold change over control bigWigs for each of these biosamples was summed using UCSC’s bigWigMerge, followed by UCSC’s bedGraphToBigWig, creating NeuN^+^ and NeuN^−^ aggregate signals. Neuron and glia b-cCREs were identified by using these bigWigs to call active b-cCREs (same as with individual ATAC experiments). Neuron-specific b-cCREs are only active based on NeuN^+^ aggregate signal and glia-specific based on NeuN^−^ aggregate signals. Shared b-cCREs are active in both NeuN^+^ and NeuN^−^ aggregate experiments, and low-signal b-cCREs are active in neither.

The *z* scores computed from these ATAC-seq experiments (bigWig files) were also used to plot the heatscatters in Fig. 2 (B and C). b-cCREs and cCREs were subset on the basis of their classifications, and each b-cCRE was plotted using its *z* score in the NeuN^+^ aggregate experiment along the *x* axis and *z* score in the NeuN^−^ aggregate experiment along the *y* axis. The plots were generated with ggplot2 in R, with the color/density being computed by the two-dimensional kernel density estimation function in the MASS library.

### Comparing b-cCREs with the putative enhancers by Yousefi *et al*. 

We downloaded putative enhancer lists DAEs and nDAEs from Yousefi *et al*. (*25*) and lifted their genomic coordinates over to GRCh38 using UCSC liftOver. We then identified DAEs and nDAEs (separately) intersecting b-cCREs within each of our defined subsets—fetal-only b-cCREs, adult-only b-cCREs, adult/fetal-shared b-cCREs, nb-cCREs, and non-cCREs using bedtools intersect (*110*). Because DAEs/nDAEs often span multiple cCREs, we used a priority order to assign them to one of five subsets: (i) any DAE intersecting a shared adult/fetal-shared b-cCRE; (ii) any DAE not in a shared b-cCRE but in a fetal b-cCRE; (iii) DAEs only in adult b-cCREs; (iv) DAEs not in any b-cCREs but in a cCRE; and (v) DAEs not in cCREs.

With each of the 10 resulting subsets of DAEs and nDAEs, we computed DNase-seq and H3K27ac and H3K4me1 ChIP-seq signals in the fetal brain across 1000 bp on either side of the DAE/nDAE center for each DAE/nDAE using deeptools pyBigWig. These signal values were aggregated across all DAE/nDAE in a subset by computing the mean at each position.

Using a similar method, we computed phyloP signal across 240 mammals (*37*), once centered on the DAE/nDAE center, and then centered on the b-cCRE/nb-cCRE center. DAEs/nDAEs on the Y chromosome and within the pseudoautosomal regions on the X chromosome were excluded from the analysis. After computing the aggregate signal for each subset, the signal was smoothed by computing a rolling average across a 21-bp window (centered with 10 bp on each side).

### Functional validation of b-cCREs using VISTA transgenic mouse assays

We downloaded the entire set of 2002 human regions tested by Pennacchio and colleagues (*49*–*51*) in vivo with transgenic mouse assays from the VISTA enhancer browser on 18 March 2024. (We discarded the 1381 mouse-only VISTA regions in our analysis.) We then used UCSC liftOver to lift these human genome coordinates (provided by VISTA in hg19) over to GRCh38; 2001 of the 2002 regions were mappable in GRCh38. We then used bedtools intersect to determine the overlap of these VISTA enhancers with cCREs.

Because the tested regions in VISTA are larger (1963 ± 1177 bp) than cCREs and often contain multiple cCREs, we developed the following method of weighting the contribution of each VISTA region to each cCRE class (adult-only b-cCREs, fetal-only b-cCREs, adult/fetal–shared b-cCREs, nb-cCREs, and non-cCREs). VISTA regions not intersecting any portion of a cCRE were assigned a score of 1 to “non-cCREs.” Any VISTA region intersecting only one cCRE was assigned a score of 1 to the class of that cCRE. If a VISTA region intersected multiple cCREs (a cCRE was allowed to partially intersect a VISTA region), then we assigned each intersection an overlap proportion based on how many base pairs of the cCRE intersected the VISTA region. We then normalize these intersection fractions in a VISTA region to sum to 1 by dividing each by the sum of all intersection proportions in the VISTA region. For example, if a VISTA region completely contained one adult-specific and one fetal-specific b-cCRE, then it would contribute a score of 0.5 to the adult and a score of 0.5 to the fetal b-cCREs. As another example, if a VISTA region completely contained one adult-specific b-cCRE and overlapped half of a nb-cCRE, then it would contribute scores of 0.67 to adult-specific b-cCREs and 0.33 to nb-cCREs.

This analysis was performed for VISTA regions based on the tissue they exhibited activity. Brain enhancers were defined as any VISTA regions with activity in forebrain, midbrain, or hindbrain tissue. Nonbrain, nervous system enhancers were defined as any VISTA regions with activity in the cranial nerve, eye, neural tube, dorsal root ganglion, or trigeminal V, but excluding those that also have activity in the aforementioned brain tissues. Non-nervous system VISTA enhancers are those with reported activity but not within the previously described tissues. Inactive VISTA regions are those that did not display activity in any tissue.

### Computing phyloP and phastCons evolutionary conservation

We determined the five biosamples cutoff of defining b-cCREs by examining evolutionary conservation of cCREs supported by various numbers of DNase-seq experiments in adult brain tissues. As described above, we computed the number of biosamples each cCRE is active in. For each cCRE, we computed the phyloP signal across 240 mammals and phastCons signal across 43 primates in a 500-bp window centered on the cCRE midpoint and then averaged over a 5-bp sliding window for smoothing. These signals computed for these cCREs were aggregated across all cCREs active in a particular number of biosamples.

We also generated a set of 10,000 control regions. These were generated by first randomly selecting 10,000 cCREs. For each of these selected cCREs, we used bedtools shuffle to identify random regions of equal size anywhere on the genome and computed the C + G mononucleotide and CpG dinucleotide content of the shuffled region. We repeated this process until we generated a random region with equal C + G and CpG content, for each of the 10,000 cCREs. We also generated phyloP-240 and phastCons-43 aggregate signals from this set of random regions to plot as a negative control. This was repeated for additional subsets of b-cCREs—fetal-specific, adult-specific, and adult/fetal-shared, as well as neuron-specific, glia-specific, and neuron/glia-shared.

### Analyzing GO using GREAT

We performed 10 rounds of GREAT analysis (*40*), each time using a cell type– or life stage–specific subset of b-cCREs as a test set and all b-cCREs as a background set. The analysis was performed using the rGREAT library (*111*). For any GO terms, such as biological process, we filtered out any term with a fold enrichment < 2 to reproduce the results of the GREAT web interface.

### Analyzing the enrichment of b-cCREs near Hi-C regions and cell type–specific marker genes

FIREs and Hi-C loops were downloaded from Hu *et al.* (*55*), and their genomic coordinates were lifted over to GRCh38 using UCSC liftOver. FIREs and loops were then intersected with b-cCRE subsets (neuron-specific, glia-specific, and neuron/glia-shared) using bedtools intersect. To calculate enrichment over control, we used bedtools shuffle to identify *N* random regions in the genome, where *N* is the number of b-cCREs in a subset of interest, and intersected those regions with FIREs/loops and computed an enrichment: number of actual intersections/number of simulated intersections. This process was repeated 100 times for each subset, and the overall enrichment was defined as the mean of the 100 enrichments. We also computed the standard deviation of those enrichments using the 100 trials.

We then compiled a list of marker genes for six brain cell types: excitatory neurons, inhibitory neurons, astrocytes, oligodendrocytes, OPCs, and microglia. For each cell type–specific marker gene, we identified the transcription start site, using GENCODE version 30 (*112*), and added 100 kb to either side to create a start/end. For each b-cCRE subset versus marker gene cell type combination, we computed the number of b-CREs within 100 kb of a marker gene using pybedtools (*113*). We then used bedtools shuffle to calculate the number of random genomic regions within 100 kb of a marker gene, which we used to calculate enrichment.

### Training VAEs to classify b-cCRE subsets

We obtained 512-bp b-cCRE–centered sequences and then performed one-hot encoding (*A* = [1, 0, 0, 0], *C* = [0, 1, 0, 0], *G* = [0, 0, 1, 0], *T* = [0, 0, 0, 1], *N* = [0.25, 0.25, 0.25, 0.25]). We then passed the one-hot–encoded sequences through a convolution layer composed of 256 filters of width 32. We then performed max pooling with a window size of 8 bp and passed the resulting convolved sequence to another convolution layer with 128 filters of length 8. The twice convolved sequences are flattened, passed through a layer of 1028 densely connected neurons before arriving at the latent space of 512 neurons. The classifier takes the latent neurons as input and passes them to an output layer of neurons equal in number to that of the number classes being predicted, i.e., 2 in the case of neurons versus glia. The decoder uses layers of densely connected neurons followed by upsampling and transpose convolution layers to reconstruct the input sequence. The VAE is simultaneously trained to minimize the reconstruction and classifier losses and the Kullback-Leibler divergence. The VAE was constructed and trained using the tensorflow.keras API (Tensorflow version 2.8) (*114*).

### Identifying enriched TF motifs using random forest classification and AME

We used the 401 TF motifs in the HOCOMOCO (v11) core catalog (https://hocomoco11.autosome.org/downloads_v11) (*64*) to identify the motifs enriched in the b-cCREs specific for each of the six single-cell types (excitatory neurons, inhibitory neurons, astrocytes, oligodendrocytes, OPCs, and microglia). Each b-cCRE was assigned a score equal to the maximum value of the position weight matrix over the b-cCRE. Negative control sequences were obtained by dinucleotide shuffling positive sequences (i.e., b-cCREs specific to a single-cell type) and similarly scored. Ten random forest classifiers were trained using the scikit-learn API (*115*). The feature importance of each motif was calculated as the average, normalized feature importance across the 10 models. AME (*116*) from the MEME suite (version 5.1) was run providing the same positive sequences and HOCOMOCO v11 motifs as input to obtain a *P* value for the enrichment of each motif in the cell type–specific b-cCREs.

### Building ChromBPNet models of chromatin accessibility

We trained ChromBPNet models to predict chromatin accessibility using the Docker image provided by the ChromBPNet authors (docker.io/kundajelab/chrombpnet:latest). We extended this Docker image to include utility functions for preprocessing data and for orchestrating jobs on a high-power computing cluster; this code is available at https://github.com/weng-lab/chrombpnet-workflow. Jobs were run on Google Cloud and a local Slurm cluster, coordinated with the Krews workflow engine (https://github.com/weng-lab/krews).

We trained models for four bulk ATAC-seq experiments from PsychENCODE: FAN-sorted neurons from VLPFC, FAN-sorted glia from VLPFC, FAN-sorted neurons from putamen, and FAN-sorted glia from putamen. We then trained models for several pseudobulk scATAC-seq datasets from adult brain. For datasets with multiple sequence alignment files in the BAM format, alignments were first pooled into a single BAM using the samtools cat function (*117*). Base pair–resolution profiles of reads at b-cCREs were then generated, bias models were trained for each dataset, and ChromBPNet models were trained for each dataset as described in the ChromBPNet wiki (https://github.com/kundajelab/chrombpnet). Last, we generated base pair–resolution importance scores for 2114-bp windows surrounding each b-cCRE for each dataset as described in the ChromBPNet wiki. All importance scores are available for visualization and download in bigWig format through PsychSCREEN.

We next build a HMM to identify sequences with a significant positive or negative influence on chromatin accessibility within each b-cCRE for each model. For all models, we extracted importance score vectors for all b-cCREs from the corresponding bigWig and discretized them into three distinct values: −1 for positions with importance *z* score < −1.96 across the whole dataset (two standard deviations below the mean), 1 for positions with *Z* > 1.96 (two standard deviations above the mean), and 0 for all other positions. We then trained a three-state HMM to recognize regions likely to emit predominantly 1s (sequences up-regulating chromatin accessibility), −1s (down-regulating chromatin accessibility), and 0s (no effect on chromatin accessibility). We found that regions positively and negatively affecting chromatin accessibility sometimes contained sequences of four or more consecutive 0s; we manually divided such regions into smaller regions by excluding these strings of consecutive 0s. The code for performing this analysis is available in https://github.com/weng-lab/chrombpnet-workflow.

### Identifying motif sites differing in importance between neurons and glia

We next used the JASPAR catalog (*118*) to identify TF motifs corresponding to the HMM-identified sites above. For each site, we extracted the sequence from the GRCh38 reference genome. We then computed a similarity score between the sequence and each motif in the JASPAR catalog. Briefly, we enumerated every possible alignment between the sequence and the motif and summed the probabilities in the motif’s position-weight matrix corresponding to the observed base on the true sequence. Optimal alignments would thus correspond to a higher similarity score. In instances where the sequence was longer than the given motif, we assumed values of 0.25 for the remaining positions, thus penalizing significant mismatches in length between the motif and the sequence. We assigned each individual site to its single most similar motif in the JASPAR catalog.

We computed motif enrichment between various pairs of motif sets identified by different sets of ChromBPNet models. For each set of models, we counted the number of instances of a given motif contained in the complete list of HMM-identified sites. We identified all motifs having at least 25 instances in each set. We then used a chi-square test to compare the fraction of motifs in each set represented by the motif in question using the chi2_contingency function in the Scipy package.

To identify differences in motif syntax between adult neurons and glia, we pooled all motif sites identified by the bulk neuron and glia models within shared b-cCREs (b-cCREs active in both neurons and glia). We then used the bedtools intersect function to identify motifs that were called as important by our HMM in neurons alone (neuron-specific motifs), glia alone (glia-specific motifs), or both neurons and glia (neuron/glia-shared motifs).

### Characterizing cell type–specific TF motif importance

Motifs in cell type–specific b-cCREs were identified using our previously developed convolutional neural network-based method (*38*). Profile scores from trained ChromBPNet models were obtained using the pyBigWig library from the deepTools package.

### LD score regression

We input our annotations, including b-cCREs and evolutionarily conserved bases, into stratified LDSC, a method for quantifying the proportion of a trait’s heritability which is causally explained by each of a series of genomic partitions (*46*). We studied heritability for a set of 204 GWAS. One hundred seventy-six of these studies were curated by the LDSC authors, many from UK Biobank and others from individually published GWAS studies. Our group obtained the remaining 28 summary statistics from the psychiatric genomics consortium. Of these, 89 GWAS are brain-related; we grouped these broadly into the following categories: ADHD, autism spectrum disorder, anorexia nervosa, behavioral traits, neuroticism, addictive disorders, bipolar disorder, schizophrenia, mood disorders, intelligence, circadian rhythm, neurostructural traits, and Alzheimer’s disease. These groupings are listed in table S4A. The remaining traits are nonbrain-related. We grouped these broadly into the following categories: immune-mediated diseases, blood cell counts, calcium homeostasis, renal function, cholestatic liver function tests, other liver function tests, lipid metabolism, vascular disease, myocardial traits, skin and hair pigmentation, cancer, and other biochemical tests.

Our analysis is conditioned on the baseline-LD model version 2.2, which contains various partitions of SNPs according to minor allele frequency, evolutionary conservation, and regulatory potential. We report heritability enrichment and associated *P* value as output by LDSC (*46*, *47*). We additionally performed random effects meta-analysis across subgroups of traits as described by Sullivan *et al.* (*48*). All analysis was performed using the Dockerized pipeline available at https://github.com/weng-lab/ldr.

Genetic correlation was computed using the “--rg” option of the “ldsc.py” script from the LDSC package. To create a list of independent traits, we first shuffled the list of all traits and then iterated the list in this shuffled order. We added each trait to the list of independent traits only if its squared genetic correlation with each of the previously added traits did not exceed 0.1. Some pairs of traits failed to run because of insufficient numbers of SNPs; we assigned these pairs correlation values of 0.

### PsychSCREEN implementation

The PsychSCREEN web portal is based on ReactJS, a flexible web application framework introduced by Facebook. ReactJS optimizes updates to the document object model, which web browsers use to represent and render web pages; this enables us to generate performant and interactive data visualizations. PsychSCREEN is written in TypeScript and uses the MaterialUI framework from Google for its user interface components. Many plots and figures in this manuscript can be reproduced directly on PsychSCREEN; they are rendered on the site as SVG and can be exported using “download” buttons found throughout the site.

PsychSCREEN’s backend is powered by a microservice architecture. Data of similar types, such as eQTLs, gene expression matrices, and b-cCREs, are each stored in a separate microservice; this improves both performance and extensibility, allowing additional data types to be integrated without disrupting the existing architecture. Data storage is primarily within Postgres databases, with some data stored in UCSC big binary formats. Microservices use GraphQL, a flexible API framework from Facebook that enables the page to retrieve precisely the data needed in each query; this greatly optimizes performance and also allows advanced users to retrieve data from PsychSCREEN programmatically. The GraphQL APIs from each microservice are combined together using the Apollo Federation framework. The PsychSCREEN API is available at https://psychscreen.api.wenglab.org/graphql; the underlying code is available at https://github.com/weng-lab/api-gateways. The PsychSCREEN website implementation is available at https://github.com/weng-lab/psychscreen.

## Supplementary Materials

### This PDF file includes:

PsychENCODE Consortium Authorship List Figs. S1 to S12 Legends for tables S1 to S6
