# Supplementary material for: Using a comprehensive atlas and predictive models to reveal the complexity and evolution of brain-active regulatory elements
Source: Sci Adv. 2024 May 23;10(21):eadj4452. doi: 10.1126/sciadv.adj4452 (PMC11114231; doi:10.1126/sciadv.adj4452)
Supplement: Supplementary file 1 — PsychENCODE Consortium Authorship List Figs. S1 to S12 Legends for tables S1 to S6 [file sciadv.adj4452_sm.pdf]

Science Advances
AAAS

Supplementary Materials for

Using a comprehensive atlas and predictive models to reveal the complexity and evolution of brain-active regulatory elements

Henry E. Pratt *et al.*

Corresponding author: Zhiping Weng, zhiping.weng@umassmed.edu

Sci. Adv. **10**, eadj4452 (2024)
DOI: 10.1126/sciadv.adj4452

The PDF file includes:

PsychENCODE Consortium Authorship List
Figs. S1 to S12
Legends for tables S1 to S6

Other Supplementary Material for this manuscript includes the following:

Tables S1 to S6

PsychENCODE Consortium Authorship List

PsychENCODE Consortium Authors

Henry Pratt[1], Nicole Shedd[1], Nishigandha Phalke[1], Matthew Jensen[2], Cindy Wen[3], Michael J. Gandal[4], Mark Gerstein[2], Daniel H. Geschwind[3], Anshul Kundaje[5], Jill E. Moore[1], Zhiping Weng[1]

PsychENCODE Consortium Collaborators

Schahram Abkarian[6], Alexej Abyzov[7], Nadav Ahituv[8], Dhivya Arasappan[9], Jose Juan Almagro Armenteros[5], Brian J. Beliveau[10], Jaroslav Bendl[6], Sabina Berretta[11], Rahul A. Bharadwaj[12], Arjun Bhattacharya[3], Lucy Bicks[3], Kristen Brennand[2], Davide Capauto[2], Frances A. Champagne[9], Tanima Chatterjee[2], Chris Chatzinakos[11], H. Isaac Chen[4], Yuhang Chen[2], Lijun Cheng[13], Yuyan Cheng[3], Andrew Chess[6], Jo-fan Chien[14], Zhiyuan Chu[2], Declan Clarke[2], Ashley Clement[8], Leonardo Collado-Torres[12], Gregory Cooper[15], Gregory Crawford[16], Rujia Dai[17], Nikolaos P. Daskalakis[11], Jose Davila-Velderrain[18], Amy Deep-Soboslay[12], Chengyu Deng[8], Christopher P. DiPietro[11], Stella Dracheva[6], Shiron Drusinsky[19], Ziheng Duan[20], Duc Duong[21], Cagatay Dursun[2], Nicholas J. Eagles[12], Jonathan Edelstien[6], Prashant S. Emani[2], John F. Fullard[6], Kiki Galani[22], Timur Galeev[2], Sophia Gaynor[13], Kiran Girdhar[6], Fernando S. Goes[23], William Greenleaf[5], Jennifer Grundman[3], Hanmin Guo[5], Qiuyu Guo[3], Chirag Gupta[24], Yoav Hadas[6], Joachim Hallmayer[5], Xikun Han[22], Vahram Haroutunian[6], Natalie Hawken[3], Chuan He[25], Ella Henry[2], Stephanie C. Hicks[26], Li-Lun Ho[22], Marcus Ho[5], Gabriel E. Hoffman[6], Yiling Huang[5], Louise A. Huuki-Myers[12], Ahyeon Hwang[20], Thomas M. Hyde[12], Artemis Iatrou[11], Fumitaka Inoue[8], Aarti Jajoo[11], Lihua Jiang[5], Peng Jin[21], Ting Jin[24], Connor Jops[4], Alexandre Jourdon[2], Riki Kawaguchi[3], Manolis Kellis[22], Saniya Khullar[24], Joel E. Kleinman[12], Steven P. Kleopoulos[6], Alex Kozlenkov[6], Arnold Kriegstein[8], Soumya Kundu[5], Cheyu Lee[20], Donghoon Lee[6], Junhao Li[14], Mingfeng Li[2], Xiao Lin[6], Chunyu Liu[17], Jason Liu[2], Jianyin Liu[3], Shuang Liu[2], Shaoke Lou[2], Jacob M. Loupe[15], Dan Lu[27], Liang Ma[28], Shaojie Ma[2], Michael Margolis[3], Jessica Mariani[2], Keri Martinowich[12], Kristen R. Maynard[12], Samantha Mazariegos[3], Ran Meng[2], Richard M. Meyers[15], Courtney Micallef[6], Tatiana Mikhailova[17], Guo-li Ming[4], Shahin Mohammadi[29], Emma Monte[5], Kelsey S. Montgomery[27], Jennifer R. Moran[13], Eran E. Mukamel[14], Angus C. Nairn[2], Charles B. Nemeroff[30], Pengyu Ni[2], Scott Norton[2], Tomasz Nowakowski[8], Larsson Omberg[27], Stephanie C. Page[12], Saejeong Park[2], Ashok Patowary[3], Reenal Pattni[5], Geo Pertea[12], Mette A. Peters[27], Dalila Pinto[6], Milos Pjanic[6], Sirisha Pochareddy[2], Katherine Pollard[8,19,31], Alex Pollen[8], Pawel F. Przytycki[19], Carolin Purmann[5], Zhaohui S. Qin[21], Ping-Ping Qu[5], Diana Quintero[3], Towfique Raj[6], Ananya S. Rajagopalan[2], Sarah Reach[6], Thomas Reimonn[1], Kerry J. Ressler[11], Deanna Ross[9], Panos Roussos[6], Joel Rozowsky[2], Misir Ruth[6], W. Brad Ruzicka[11], Stephan J. Sanders[8,32], Juliane M. Schneider[27], Soraya Scuderi[2], Robert Sebra[6], Nenad Sestan[2], Nicholas Seyfried[21], Zhiping Shao[6], Annie W. Shieh[33], Joo Heon Shin[12], Mario Skarica[2], Clara Snijders[11], Hongjun Song[4], Matthew W. State[8], Jason Stein[34], Marilyn Steyert[8], Sivan Subburaju[11], Thomas Sudhof[5], Michael Synder[5], Ran Tao[12], Karen Therrien[6], Li-Huei Tsai[22], Alexander E. Urban[5], Flora M. Vaccarino[2], Harm van Bakel[6], Daniel Vo[4], Georgios Voloudakis[6], Brie Wamsley[3], Daifeng Wang[24], Sidney H. Wang[33], Tao Wang[5], Yifan Wang[7], Jonathan Warrell[2], Yu Wei[17], Annika K. Weimer[5], Daniel R. Weinberger[12], Sean Whalen[19], Kevin P. White[35], A. Jeremy Willsey[8], Hyejung Won[34], Wing Wong[5], Feinan Wu[2], Hao Wu[21], Stefan Wuchty[36], Dennis Wylie[9], Siwei Xu[20], Chloe X. Yap[37], Zane R. Zeier[36], Biao Zeng[6], Bin Zhang[6], Chunling Zhang[17], Jing Zhang[20], Pan Zhang[3], Yanqiong Zhang[34], Xiao Zhou[2], Ryan Ziffra[8], Trisha M. Zintel[27]

[1]University of Massachusetts Chan Medical School, Worcester, MA, USA. [2]Yale University, New Haven, CT, USA. [3]University of California, Los Angeles, Los Angeles, CA, USA. [4]University of Pennsylvania, Philadelphia, PA, USA. [5]Stanford University, Stanford, CA, USA. [6]Icahn School of Medicine at Mount Sinai, New York, NY, USA. [7]Mayo Clinic Rochester, Rochester, MN, USA. [8]University of California, San Francisco, San Francisco, CA, USA. [9]The University of Texas at Austin, Austin, TX, USA. [10]University of Washington, Seattle, WA, USA. [11]McLean Hospital, Harvard Medical School, Belmont, MA, USA. [12]Lieber Institute for Brain Development, Baltimore, MD, USA. [13]Tempus Labs, Inc., Chicago, IL, USA. [14]University of California, San Diego, San Diego, CA, USA. [15]HudsonAlpha Institute for Biotechnology, Huntsville, AL, USA. [16]Duke University, Durham, NC, USA. [17]SUNY Upstate Medical University, Syracuse, NY, USA. [18]Human Technopole, Milan, Italy. [19]Gladstone Institutes, San Francisco, CA, USA. [20]University of California, Irvine, Irvine, CA, USA. [21]Emory University, Atlanta, GA, USA. [22]Massachusetts Institute of Technology, Cambridge, MA, USA. [23]Johns Hopkins University, Baltimore, MD, USA. [24]University of

Wisconsin-Madison, Madison, WI, USA. [25]The University of Chicago, Chicago, IL, USA. [26]Johns Hopkins Bloomberg School of Public Health, Baltimore, MD, USA. [27]Sage Bionetworks, Seattle, WA, USA. [28]The University of Texas Health Science Center at San Antonio, San Antonio, TX, USA. [29]Broad Institute of MIT and Harvard, Cambridge, MA, USA. [30]The University of Texas at Austin Dell Medical School, Austin, MA, USA. [31]Chan Zuckerburg Biohub San Francisco, San Francisco, CA, USA. [32]University of Oxford, Oxford, England, UK. [33]The University of Texas Health Science Center at Houston, Houston, TX, USA. [34]University of North Carolina at Chapel Hill, Chapel Hill, USA. [35]National University of Singapore, Singapore, Singapore. [36]University of Miami, Miami, FL, USA. [37]University of Queensland, Queensland, NZ.

Pratt, Andrews, Shedd, fig. S1

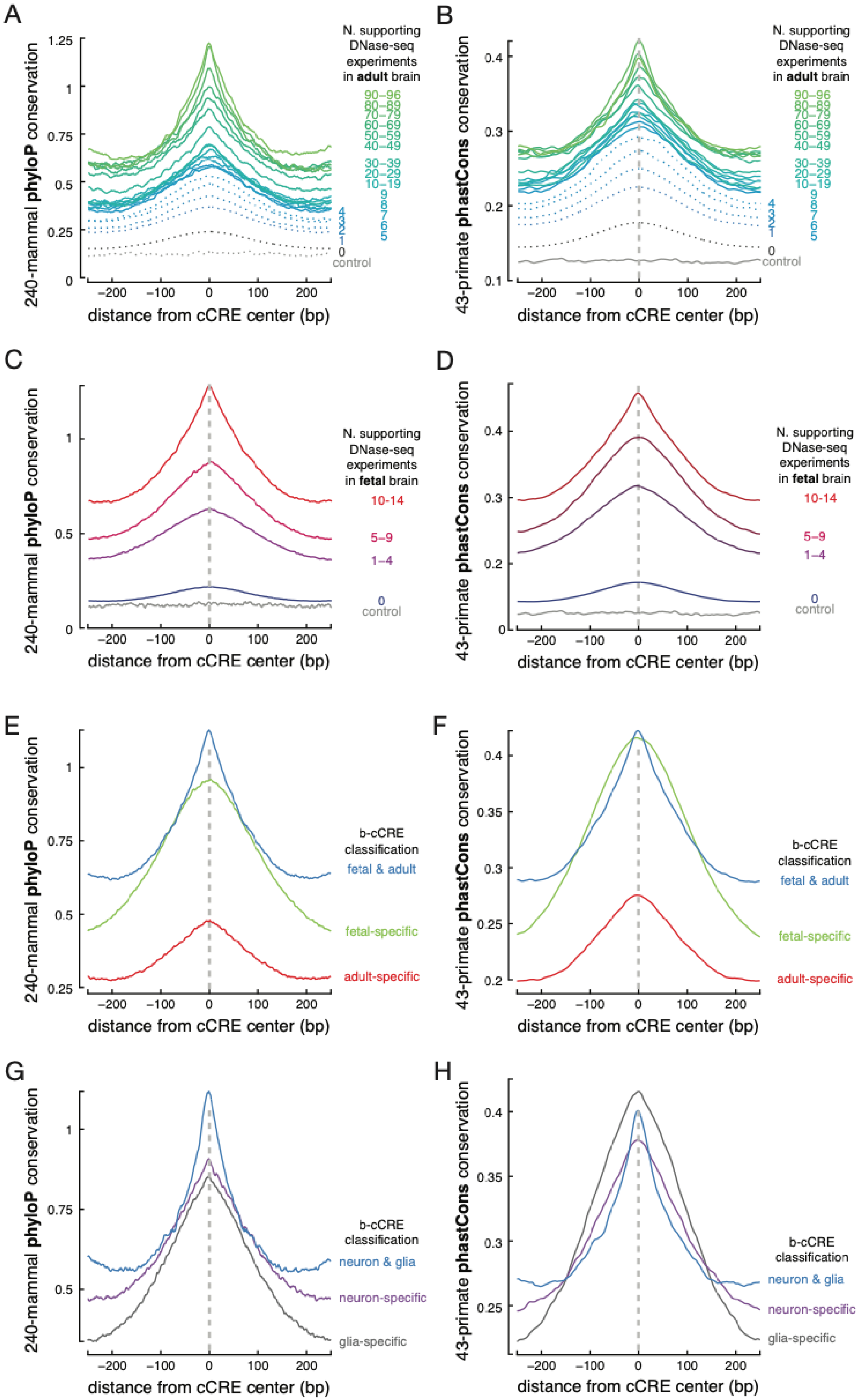

Fig. S1. Mammalian and Primate Conservation of b-cCRE subsets

Conservation scores of candidate cis-regulatory elements (cCREs), binned based on cCRE/b-cCRE subsets. Scores of 500 bp around each cCRE center are computed, then averaged for all cCREs in a particular subset.

A. 240-mammal phyloP scores, binned by activity in ENCODE adult brain biosamples

B. 43-primate phastCons scores, binned by activity in ENCODE adult brain biosamples.

C. 240-mammal phyloP scores, binned by activity in ENCODE fetal brain biosamples

D. 43-primate phastCons scores, binned by activity in ENCODE fetal brain biosamples.

E. 240-mammal phyloP scores, binned by activity in fetal and/or adult brain biosamples

F. 43-primate phastCons scores, binned by activity in fetal and/or adult brain biosamples

G. 240-mammal phyloP scores, binned by activity in neuron and/or glia biosamples

H. 43-primate phastCons scores, binned by activity in neuron and/or glia biosamples

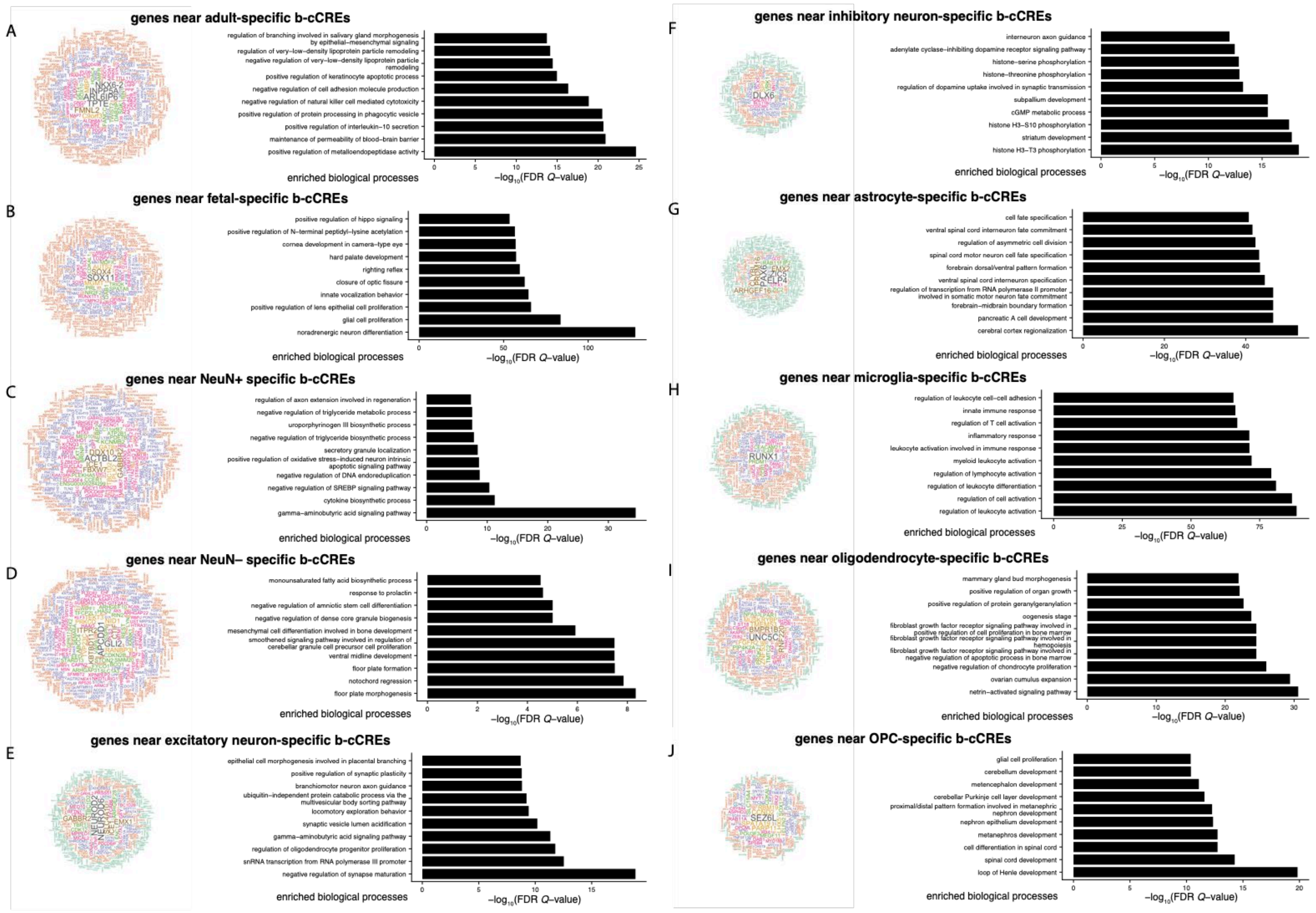

Fig. S2. GREAT gene ontology analysis of cell-type and age-specific b-cCRE subsets

GREAT analysis of cell type and age-specific sets of b-cCREs, against all b-cCREs as a background.

A. adult-specific

B. fetal-specific

C. neuron-specific

D. glia-specific

E. excitatory neuron-specific

F. inhibitory neuron-specific

G. astrocyte-specific

H. microglia-specific

I. oligodendrocyte-specific

J. oligodendrocyte precursor (OPC)-specific

In each panel, the word cloud on the left displays 400 Ensembl genes, with the font size and color indicating the p-value of the gene. Bar plots on the right display the p-values of the 10 most significantly enriched biological processes.

Pratt, Andrews, Shedd, fig. S3

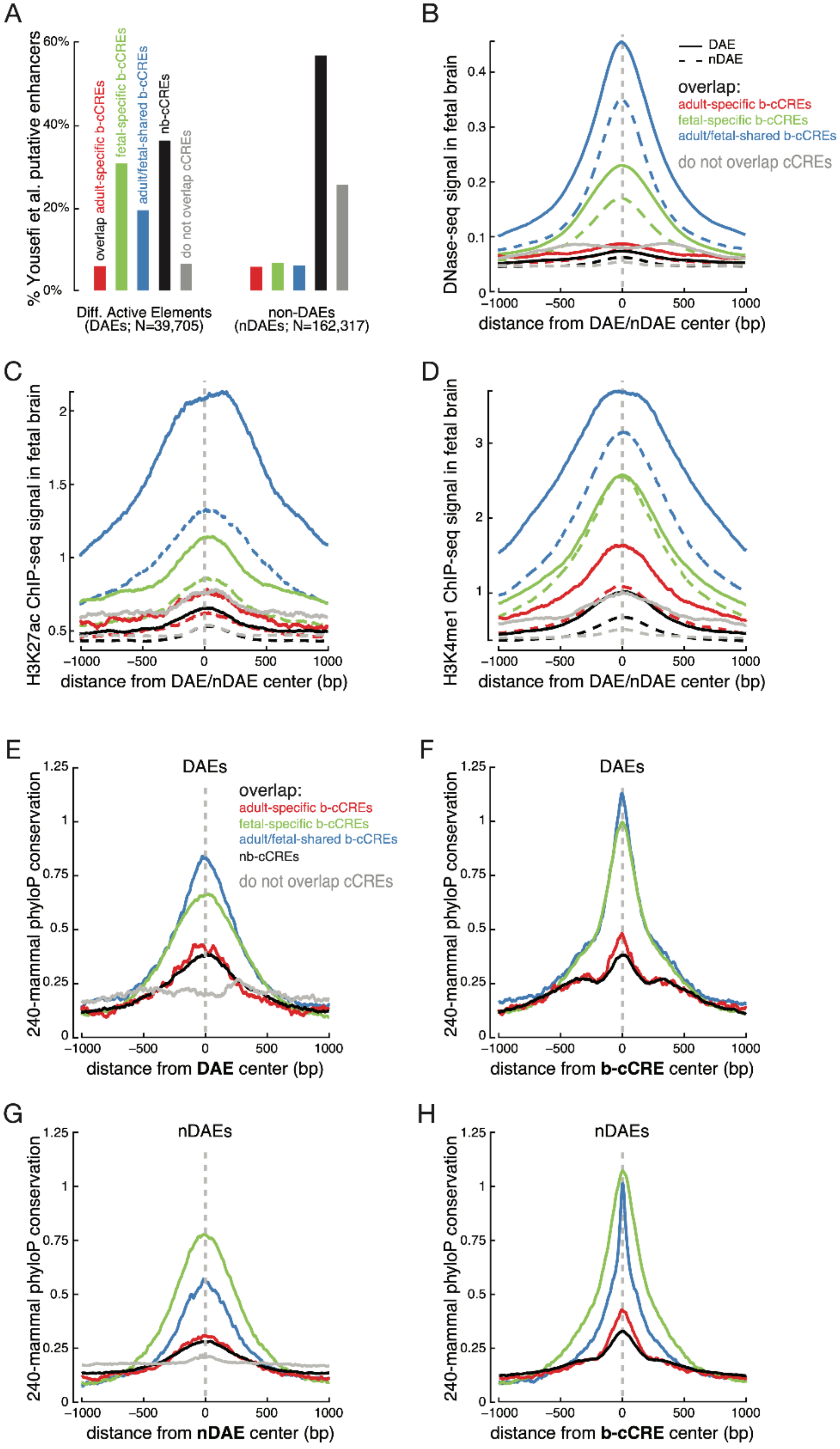

Fig. S3. Comparison of b-cCREs with DAEs from *Yousefi et al. (25)*

A. Bar chart displaying percentage of Yousefi et al. (*25*) putative enhancers intersecting with subsets of b-cCREs (adult specific in red, fetal-specific in green, and adult/fetal-shared in blue), non-brain cCREs (black), and non-cCREs (gray). Overlap with differentially active elements (DAEs) is on the left, overlap with non-differentially active elements (nDAEs) is on the right.
B. DNase-seq signal in fetal brain tissue, aggregated across all DAEs or nDAEs overlapping b-cCRE subsets.
C. H3K27ac ChIP signal in fetal spinal cord tissue, aggregated across all DAEs or nDAEs overlapping b-cCRE subsets.
D. H3K4me1 ChIP signal in fetal brain tissue, aggregated across all DAEs or nDAEs overlapping b-cCRE subsets.
E. 240-mammal phyloP conservation, aggregated across all DAEs overlapping adult-specific b-cCREs (red), fetal-specific b-cCREs (green), adult/fetal-shared b-cCREs (blue), non-brain cCREs (black), and non-cCREs (gray).
F. 240-mammal phyloP conservation, aggregated across all b-cCREs – adult-specific b-cCREs (red), fetal-specific b-cCREs (green), adult/fetal-shared b-cCREs (blue), non-brain cCREs (black) – intersecting DAEs.
G. 240-mammal phyloP conservation, aggregated across all nDAEs overlapping adult-specific b-cCREs (red), fetal-specific b-cCREs (green), adult/fetal-shared b-cCREs (blue), non-brain cCREs (black), and non-cCREs (gray).
H. 240-mammal phyloP conservation, aggregated across all b-cCREs – adult-specific b-cCREs (red), fetal-specific b-cCREs (green), adult/fetal-shared b-cCREs (blue), non-brain cCREs (black) – intersecting nDAEs.

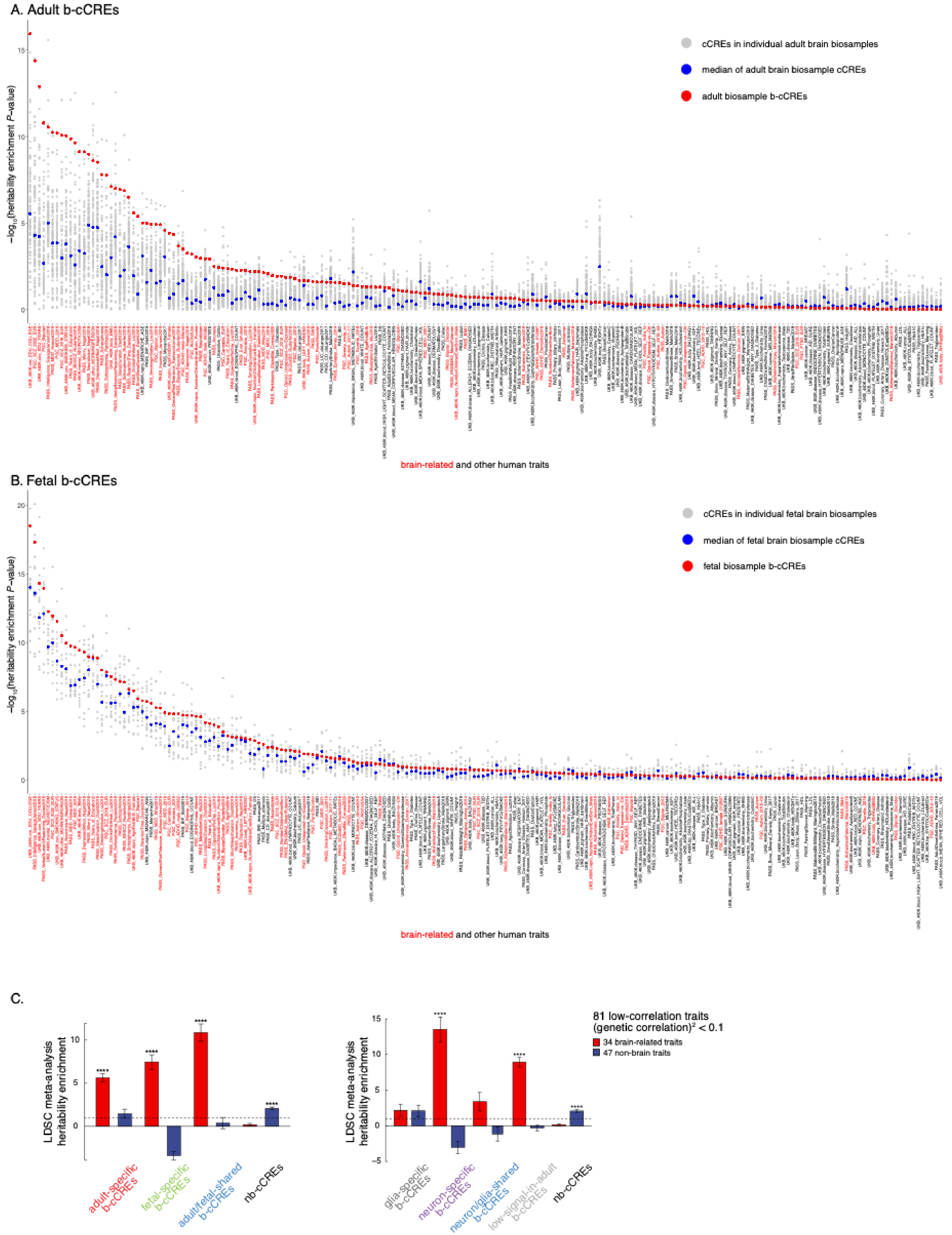

Fig. S4. LDSC of b-cCREs and cCREs active in brain biosamples

A. LD-score regression and partitioned heritability analysis of individual brain experiments (gray) and selected b-cCREs (red), as well as the median of each individual experiment (blue) against 204 GWAS summary statistics, including 87 brain and psychiatric-related studies (red) in adult biosamples.
B. LD-score regression and partitioned heritability analysis of individual brain experiments (gray) and selected b-cCREs (red), as well as the median of each individual experiment (blue) against 204 GWAS summary statistics, including 87 brain and psychiatric-related studies (red) in fetal biosamples.
C. Heritability enrichment meta-analysis of adult-specific, fetal-specific, shared b-cCREs, and nb-cCREs (left) and neuron-specific, glia-specific, neuron/glia-shared b-cCREs, b-cCREs with low signal in both NeuN+ and NeuN– adult samples, and nb-cCREs (right) in brain-related traits (red) and non-brain-related traits (blue) with low genetic correlation. LDSC meta-analysis *P*-value for enrichment in heritability of genetic variants residing in subsets of b-cCREs: * $P < 0.05$, ** $P < 0.01$, *** $P < 0.001$, **** $P < 0.0001$.

Pratt, Andrews, Shedd, fig. S5

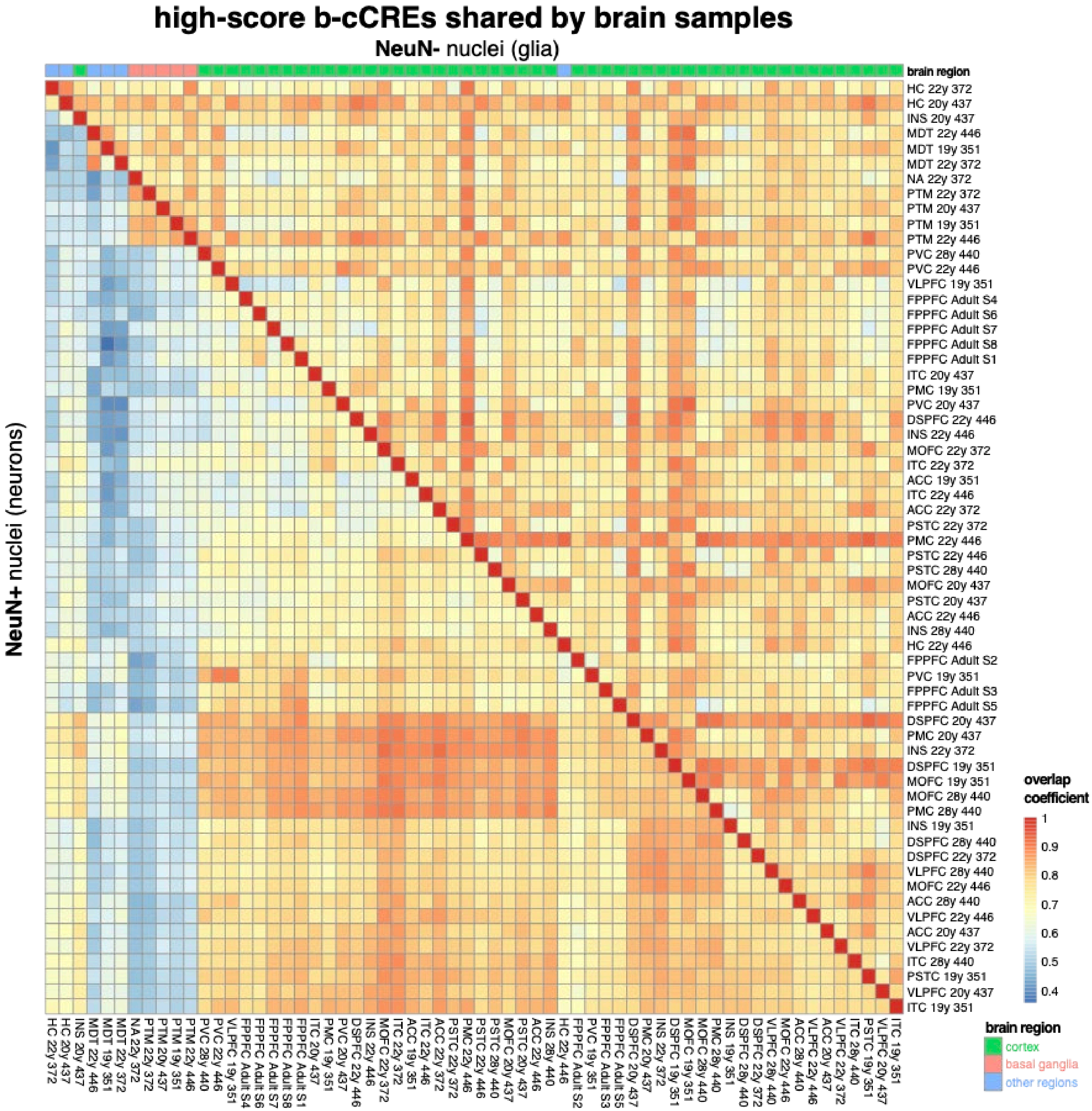

Fig. S5. Overlap of active b-cCREs between different brain regions

Heatmap illustrating the overlap coefficient between active b-cCREs from individual ATAC biosamples, sorted by hierarchical clustering. The bottom-left corner displays the overlap between FAN-sorted NeuN+ biosamples, while the top-right corner displays the overlap between matching FAN-sorted NeuN- biosamples. The colorbar along the top identifies the broad brain region category of each sample—cerebral cortex (green), basal ganglia (red), or other regions (blue).

Pratt, Andrews, Shedd, fig. S6

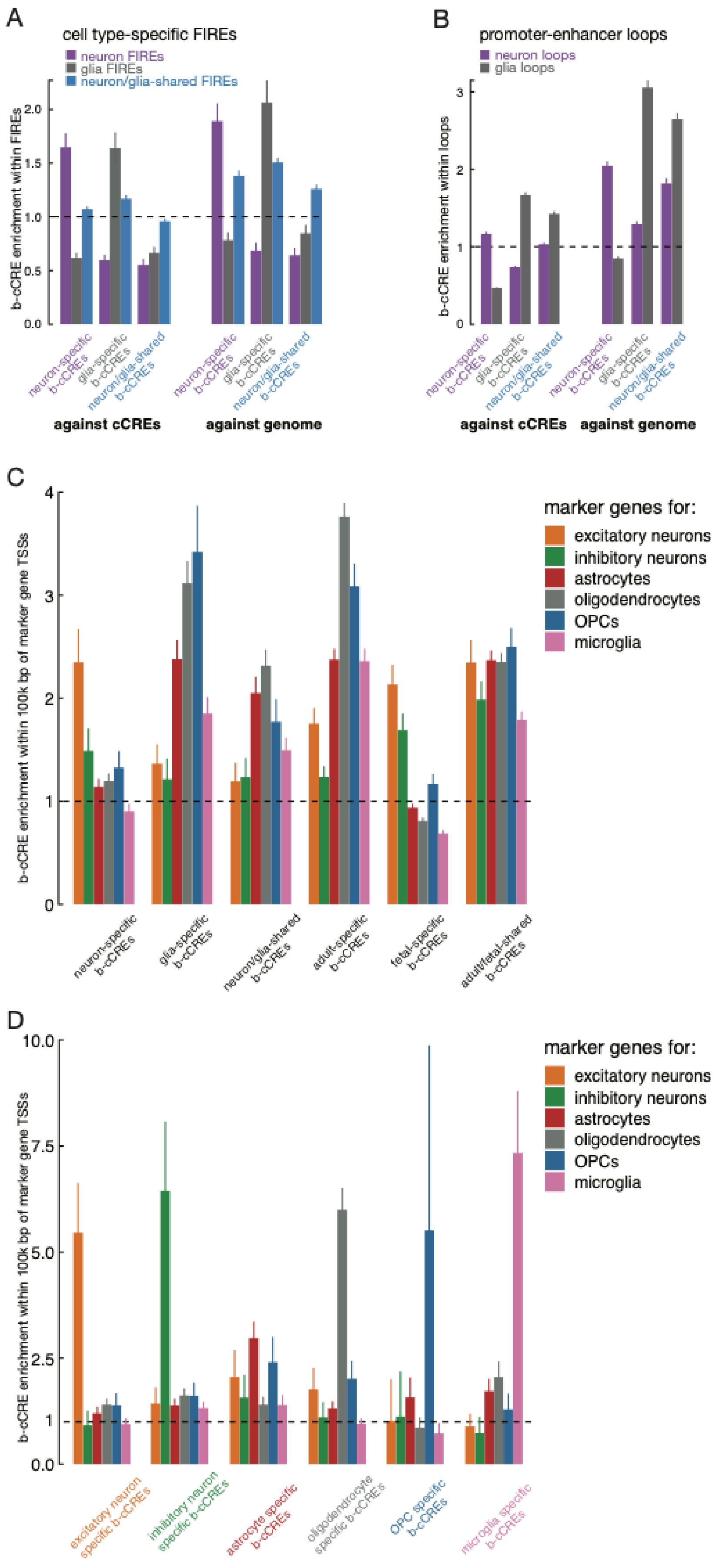

Fig. S6. Enrichment of b-cCRE subsets within published regulatory regions

A. Bar plot of b-cCRE enrichment within frequently interacting regions (FIREs) derived from Hi-C data in FAN-sorted neurons and glia from the adult brain. Compares enrichment of (from left to right) neuron-specific, glia-specific, and neuron/glia-shared b-cCREs within neuron-specific (purple), glia-specific (gray), and neuron/glia common (blue) FIREs against cCREs (left panel) and against genomic regions (right panel).
B. Bar plot of b-cCRE enrichment within promoter-enhancer loops derived from Hi-C data in FAN-sorted neurons and glia from the adult brain. Compares enrichment of (from left to right) neuron-specific, glia-specific, and neuron/glia-shared b-cCREs within neuron (purple) and glia (gray) promoter-enhancer loops against cCREs (left panel) and against genomic regions (right panel).
C. Bar plot of b-cCRE enrichment within 100k bp of marker gene transcription start sites (TSSs). Compares enrichment of (from left to right) neuron-specific, glia-specific, neuron/glia-shared, adult-specific, fetal-specific, and adult b-cCREs within excitatory neuron (orange), inhibitory neuron (green), astrocyte (red), oligodendrocyte (gray), OPC (blue), and microglia (pink) specific marker genes.
D. Bar plot of b-cCRE enrichment within 100k bp of marker gene transcription start sites (TSSs). Compares enrichment of (from left to right) excitatory neuron, inhibitory neuron, astrocyte, oligodendrocyte, OPC and microglia specific b-cCREs within excitatory neuron (orange), inhibitory neuron (green), astrocyte (red), oligodendrocyte (gray), OPC (blue), and microglia (pink) specific marker genes.

Pratt, Andrews, Shedd, fig. S7

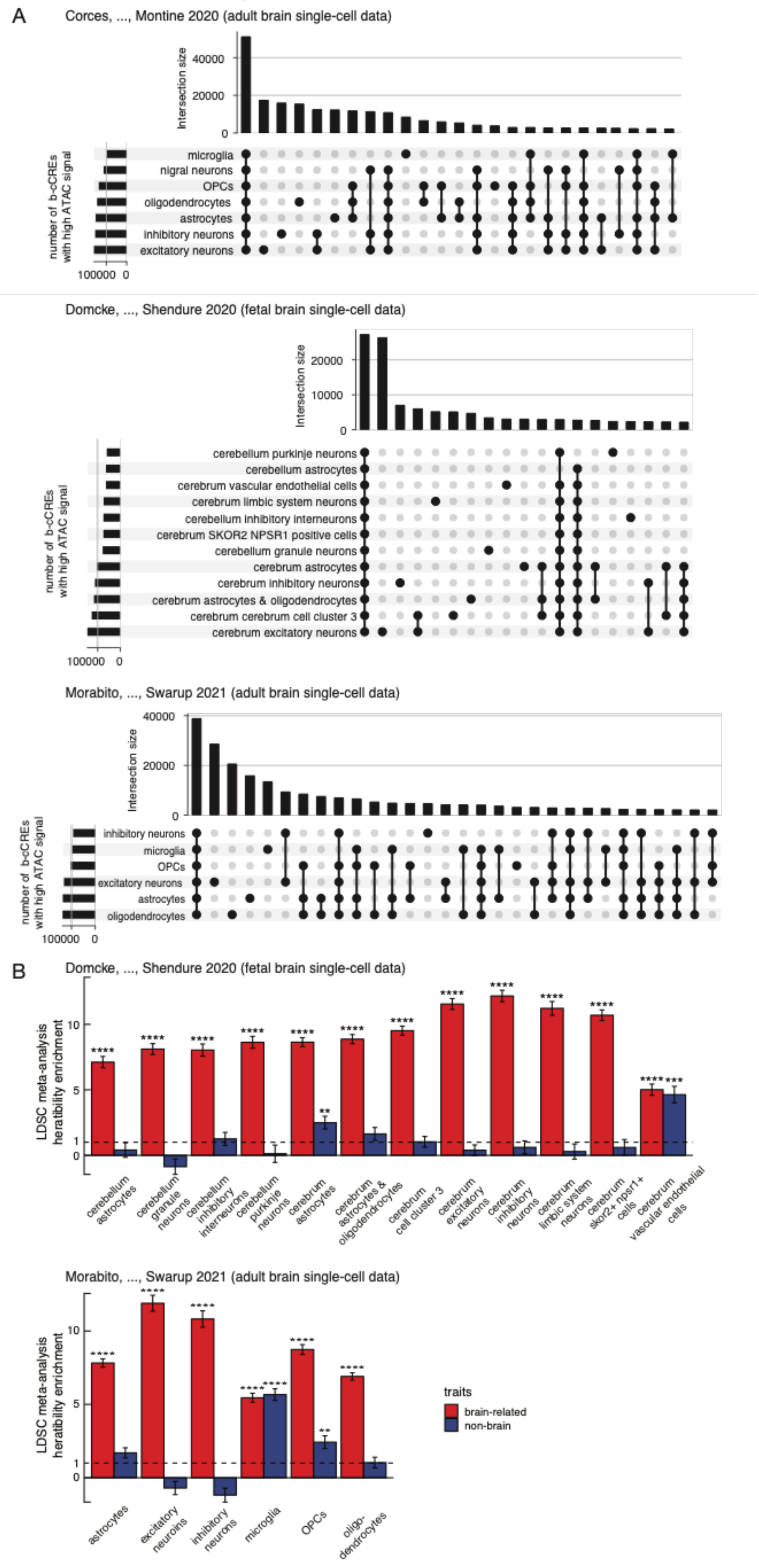

Fig. S7. Identification of cell-type-specific b-cCREs

A. UpSet plot of active b-cCREs in each brain cell type from three single-cell ATAC-seq studies. For each study, the lower-left bar plot shows the total numbers of b-cCREs with high signal in the pseudo-bulk ATAC-seq data generated for each cell type. Only intersections with more than 2000 ATAC peaks are included in the UpSet plots.

B. Heritability enrichment meta-analysis of brain cell-type active b-cCREs in brain-related (red) and non-brain-related (blue) from two single-cell ATAC-seq studies. LDSC meta-analysis *P*-value for enrichment in heritability of genetic variants residing in subsets of b-cCREs: * $P < 0.05$, ** $P < 0.01$, *** $P < 0.001$, **** $P < 0.0001$.

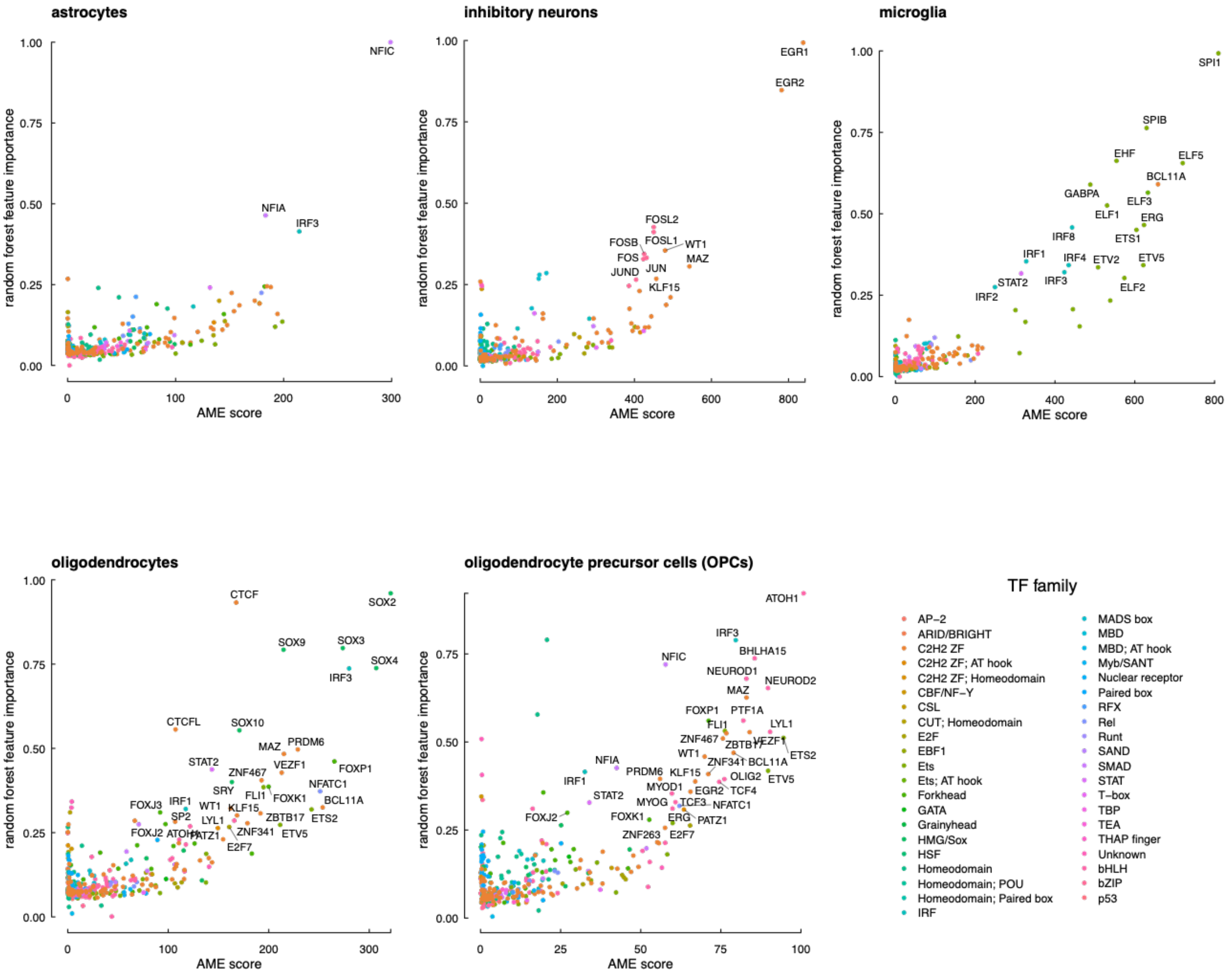

Fig. S8. Transcription factor motifs identified within cell-type specific b-cCREs

Comparison of Analysis of Motif Enrichment (AME) scores (x-axis) and random forest feature importance (y-axis), to identify transcription factor motifs that are found within cell-type specific b-cCRE subsets, colored by the transcription factor family. Each scatter plot represents a different cell type from Corces et al. (*19*) single-cell data:

Top left: Astrocytes
Top middle: Inhibitory Neurons
Top right: Microglia
Bottom left: Oligodendrocytes
Bottom middle: Oligodendrocyte Precursors (OPCs)

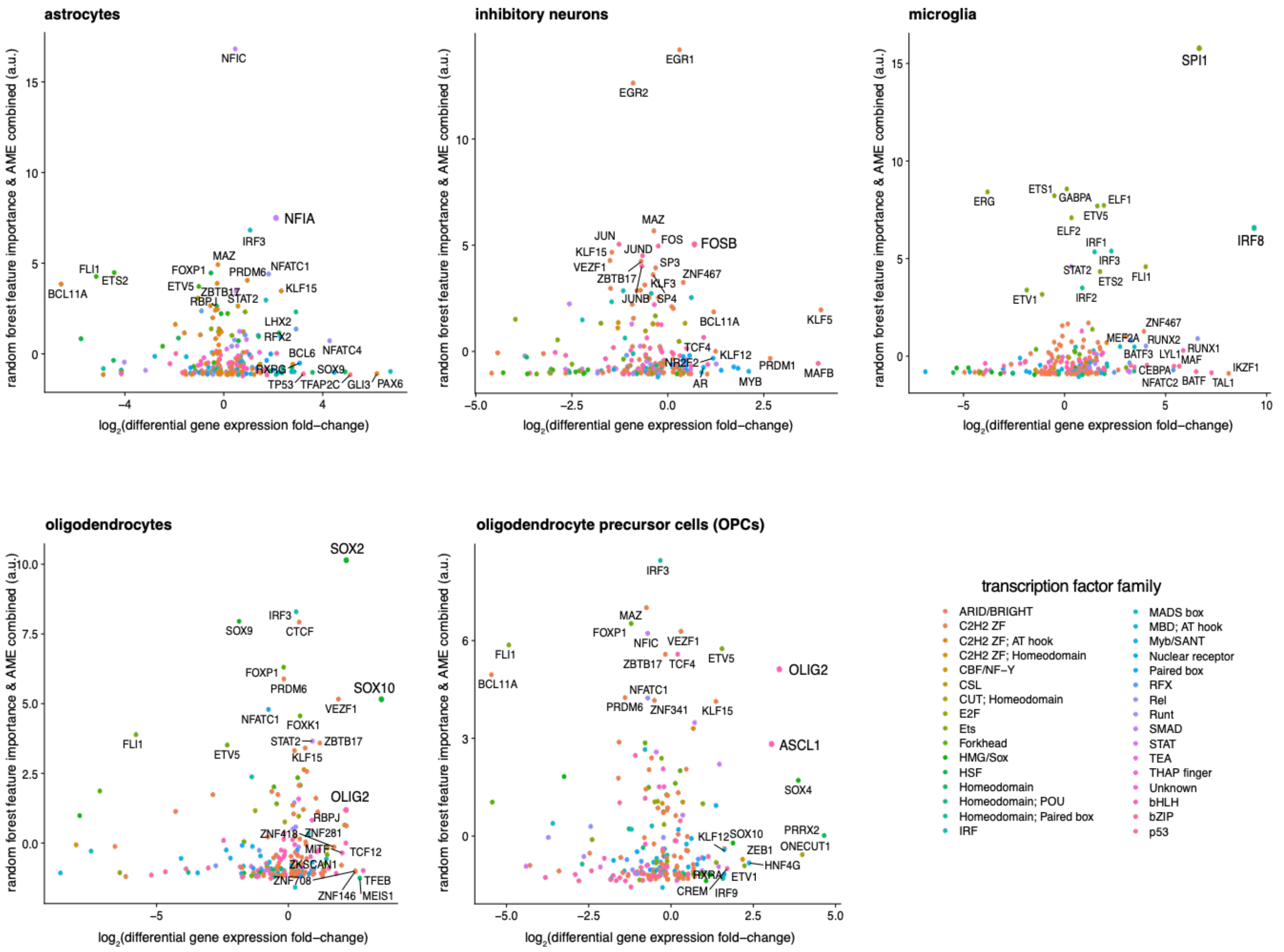

Fig. S9. Differential Gene Expression of Transcription Factors against RF feature importance and AME results
Differential Gene Expression in each cell type against all other cell types (x-axis) against sum of scaled random forest feature importance and Analysis of Motif Enrichment (AME) scores using arbitrary units (a.u.) (y-axis), colored by transcription factor family. Each scatter plot represents a different cell type from Corces et al. (*19*) single-cell data:

Top left: Astrocytes
Top middle: Inhibitory Neurons
Top right: Microglia
Bottom left: Oligodendrocytes
Bottom middle: Oligodendrocyte Precursor Cells

Pratt, Andrews, Shedd, fig. S10

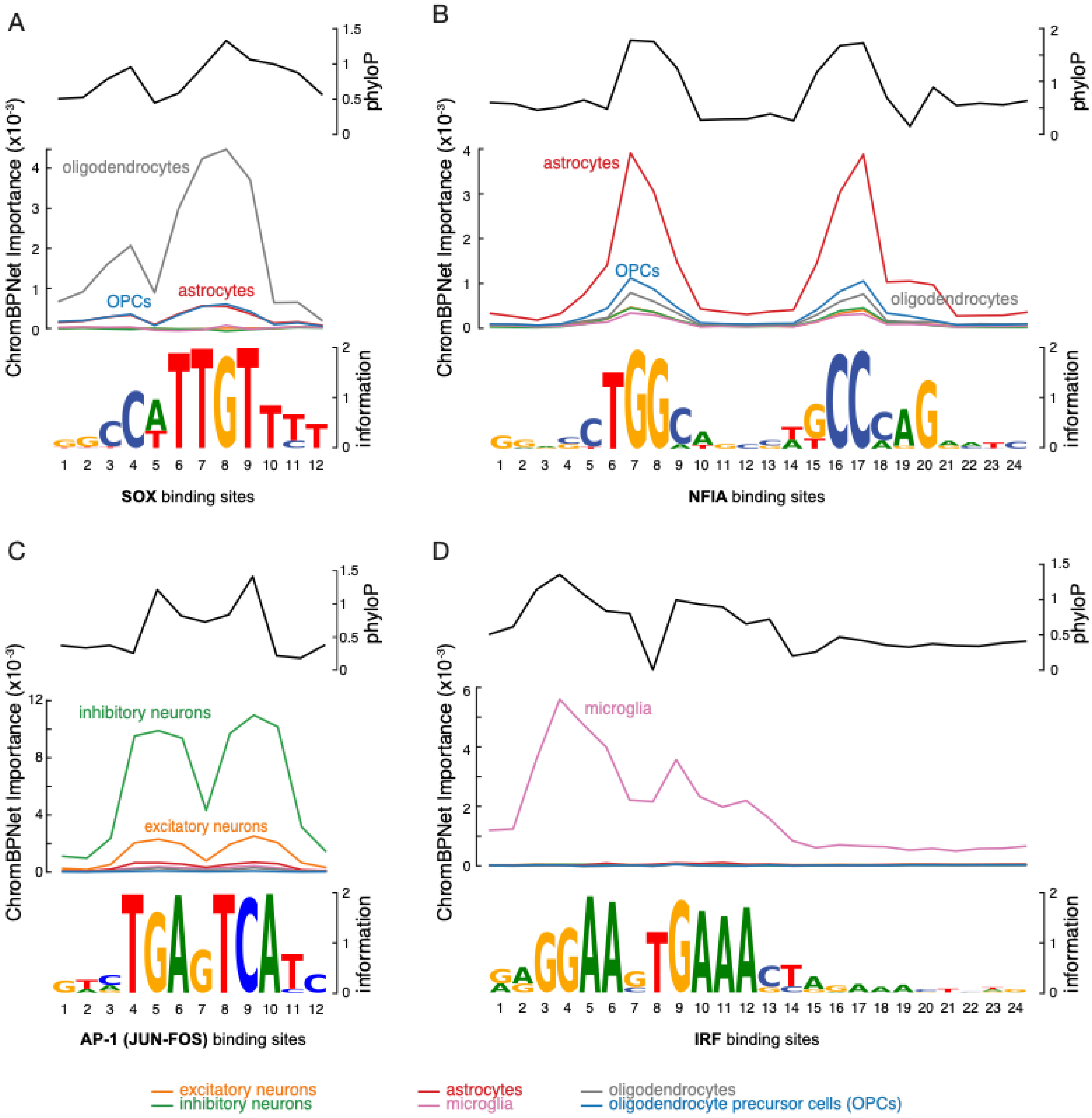

Fig. S10. ChromBPNet importance scores of deep-learned TF motifs in individual brain cell types

Average ChromBPNet importance scores of de-novo discovered (A) SOX, (B) NFIA, (C) AP-1 (JUN-FOS), and (D) IRF binding sites from trained single-cell ChromBPNet models in each cell type as colored, along with average phyloP score at those binding sites.

Pratt, Andrews, Shedd, fig. S11

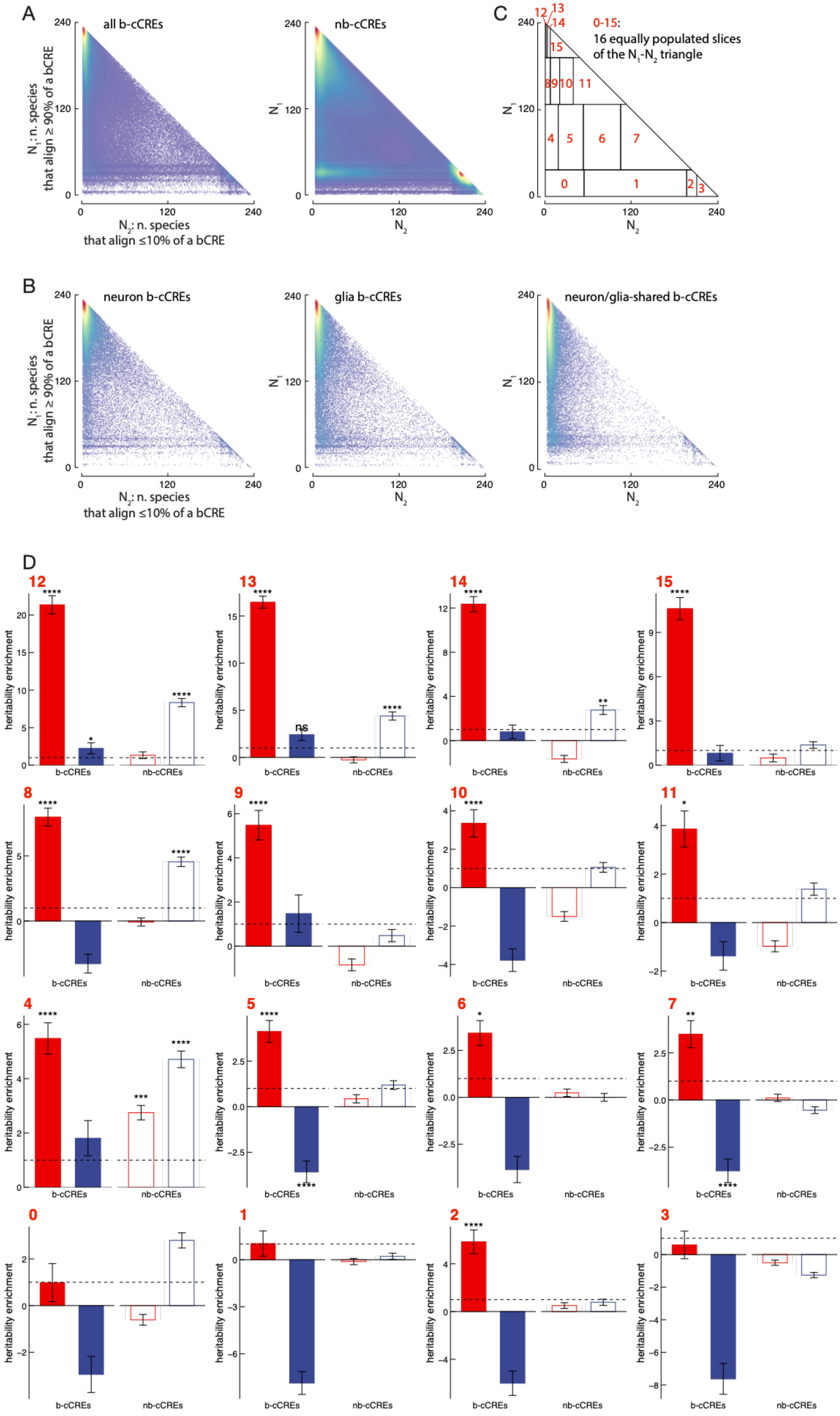

Fig. S11. LDSC of b-cCREs within 16 bins defined based on mammalian conservation

A. Evolutionary conservation of b-cCREs (left) and nb-cCREs (right) according to the mammalian genomes in which they align. N_1 indicated the number of genomes aligning ≥ 90% of the b-cCRE's sequence. N_2 denotes the number of genomes aligning ≤ 10% of the b-cCRE's sequence.

B. Evolutionary conservation of adult-specific (left), fetal-specific (middle), and adult/fetal-shared (right) b-cCREs according to the number of mammalian genomes in which they align.

C. Schematic of how the triangle can be divided into 16 equally populated slices.

D. Heritability enrichment meta-analysis of b-cCREs and nb-cCREs intersecting each of these 16 slices in brain-related (red) and non-brain-related (blue) traits. LDSC meta-analysis *P*-value for enrichment in heritability of genetic variants residing in subsets of b-cCREs: * $P < 0.05$, ** $P < 0.01$, *** $P < 0.001$, **** $P < 0.0001$.

Pratt, Andrews, Shedd, fig. S12

Corces, ..., Montine 2020 (adult brain single-cell ATAC-seq data)   0-15: 16 equally populated slices of the N_1-N_2 mammalian-genome-alignment triangle

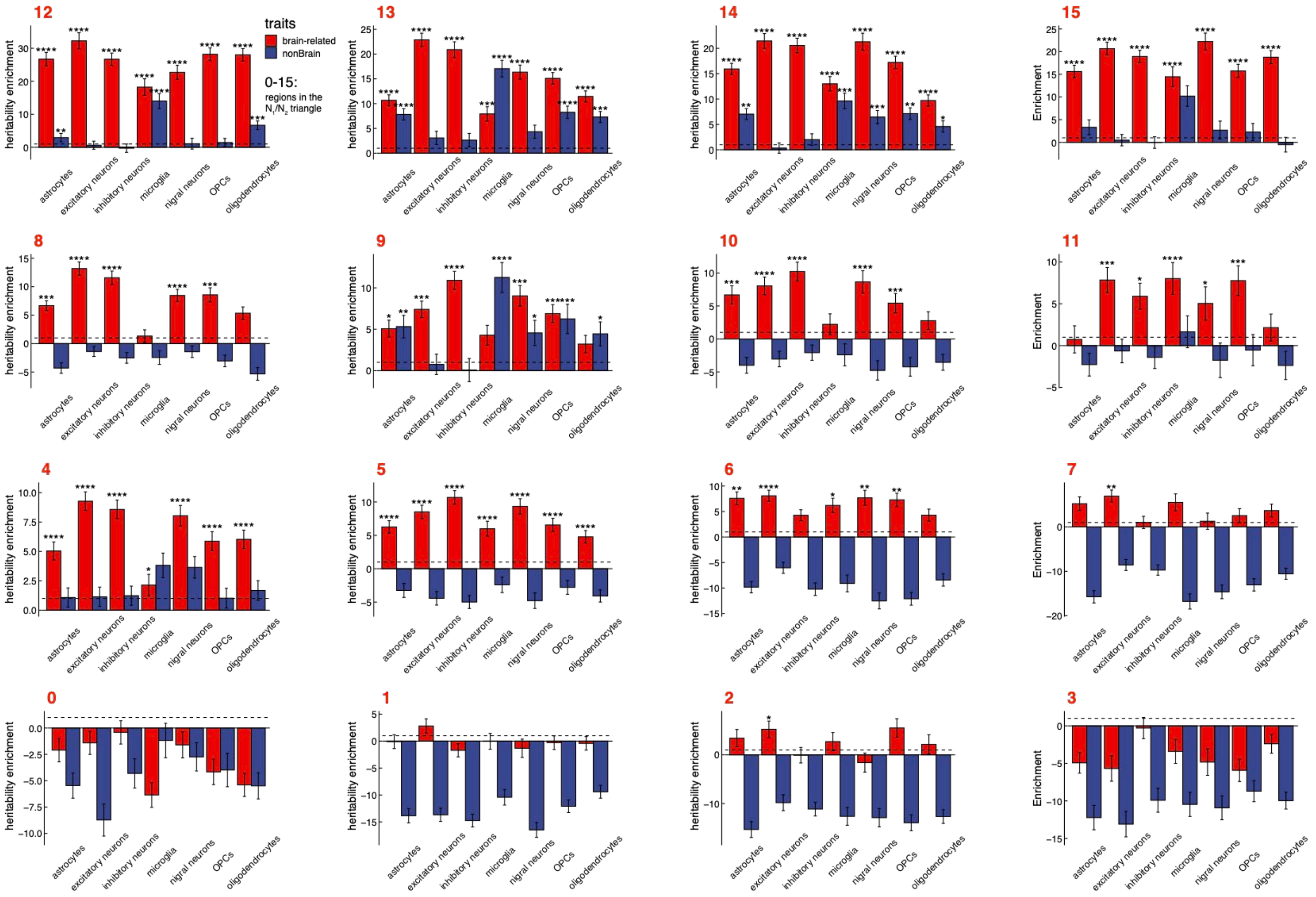

Fig. S12. LDSC of cell-type specific b-cCREs

Heritability enrichment meta-analysis of cell-type specific b-cCREs from Corces et al. (*19*), divided into 16 sections based on evolutionary conservation (as in **Fig. S8**) in brain-related (red) and non-brain-related (blue) traits. LDSC meta-analysis *P*-value for enrichment in heritability of genetic variants residing in subsets of b-cCREs: * $P < 0.05$, ** $P < 0.01$, *** $P < 0.001$, **** $P < 0.0001$.

Other Supplementary Material for this manuscript includes the following:

Table S1 - Publicly available datasets used in analysis
A. ENCODE DNase biosamples used to generate adult b-cCRE list.
B. ENCODE DNase biosamples used to generate fetal b-cCRE list.
C. ENCODE/psychENCODE fetal brain experiments used for comparison against Yousefi et al. (*25*) DAEs.
D. Publicly available FAN-sorted bulk ATAC-seq data used in analysis.
E. Marker genes curated from publicly available data.
F. Publicly available single-cell ATAC-seq data used in analysis.
G. Functional validation of cCREs using the human genomic regions tested by transgenic mouse assays in the VISTA database.

Table S2 - b-cCRE zscores and classifications within different cell types and developmental time points
A. Computed z-scores of b-cCREs. NeuN+ and NeuN- scores are the z-scores from aggregated bigWigs of NeuN FANs sorted ATAC data, and single-cell scores are pseudo-bulk z-scores from Corces et. al. (*19*) adult and fetal b-cCRE scores are computed from the average z-score of all ENCODE DNase biosamples.
B. b-cCRE classifications for each subset.TRUE/FALSE assignments depending on whether a b-cCRE is called within that subset (see Methods).

Table S3 - Results from GREAT gene ontology analysis
GREAT Gene Ontology analysis of cell type- or age-specific b-cCREs against all b-cCREs as a background
A. adult-specific
B. fetal-specific
C. neuron-specific
D. glia-specific
E. excitatory neuron-specific
F. inhibitory neuron-specific
G. astrocyte-specific
H. microglia-specific
I. oligodendrocyte-specific
J. oligodendrocyte precursor (OPC)-specific
S3, A to J have 2 tabs: the first with Ensembl genes near that b-cCRE subset, and the second with gene ontology biological processes enriched in those nearby genes.

Table S4 - LDSC analysis and meta-analysis results
A. 204 traits used in this study and their classification (brain/non-brain). The subset of 81 traits with pair-wise squared genetic correlations less than 0.1 is indicated in the last column.
B. Genetic correlation between the summary statistics between the 204 GWAS.
C. LDSC analysis of adult b-cCREs and active cCREs from individual adult brain biosamples (described in Table S1A).
D. LDSC analysis of fetal b-cCREs and active cCREs from individual fetal brain biosamples (described in Table S1B).
E. LDSC meta-analysis on the fetal-specific, adult-specific, and adult/fetal-shared subsets of b-cCREs.
F. LDSC meta-analysis on the subset of 81 traits with pair-wise squared genetic correlation less than 0.1.
G. LDSC meta-analysis on the neuron-specific, glia-specific, and neuron/glia-shared subsets of b-cCREs.
H. LDSC meta-analysis on the Corces-Montine study.
I. LDSC meta-analysis on the Morabito-Swarup study.

J. LDSC meta-analysis on the Domcke-Shendure study.
K. LDSC meta-analysis on the subsets of b-cCREs classified by evolutionary group and fetal / adult.
L. LDSC meta-analysis split by evolutionary origin (non-primates, plus primate clades).

Table S5 - Results of Random Forest Feature Importance (RF), Analysis of Motif Enrichment (AME), and differential gene expression (DEG) analyses.
A. Differential expression of Excitatory Neurons against all other cell types from scRNA-seq data.
B. Random Forest Feature Importance and AME enrichment scores from sequence of Excitatory Neuron-specific b-cCREs.
C. Differential expression of Astrocytes against all other cell types from scRNA-seq data.
D. Random Forest Feature Importance and AME enrichment scores from sequence of Astrocyte-specific b-cCREs.
E. Differential expression of Inhibitory Neurons against all other cell types from scRNA-seq data.
F. Random Forest Feature Importance and AME enrichment scores from sequence of Inhibitory Neuron-specific b-cCREs.
G. Differential expression of Microglia against all other cell types from scRNA-seq data.
H. Random Forest Feature Importance and AME enrichment scores from sequence of Microglia-specific b-cCREs.
I. Differential expression of Oligodendrocytes against all other cell types from scRNA-seq data.
J. Random Forest Feature Importance and AME enrichment scores from sequence of Oligodendrocyte-specific b-cCREs.
K. Differential expression of OPCs against all other cell types from scRNA-seq data.
L. Random Forest Feature Importance and AME enrichment scores from sequence of OPC-specific b-cCREs.

Table S6 - Bias factorized ChromBPNet training and quality check report metrics
A. Bias model performance in peaks from bias model training report.
B. ChromBPNet model performance in peaks from ChromBPNet model training report.
